# Classification and Verification of Handwritten Signatures with Time Causal Information Theory Quantifiers

**DOI:** 10.1371/journal.pone.0166868

**Published:** 2016-12-01

**Authors:** Osvaldo A. Rosso, Raydonal Ospina, Alejandro C. Frery

**Affiliations:** 1 Instituto de Física, Universidade Federal de Alagoas (UFAL), Maceió, AL, Brazil; 2 Instituto Tecnológico de Buenos Aires (ITBA), and CONICET, Ciudad Autónoma de Buenos Aires, Argentina; 3 Facultad de Ingeniería y Ciencias Aplicadas, Universidad de los Andes, Santiago, Chile; 4 Centro de Ciências Exatas e da Natureza, Departamento de Estatística, Universidade Federal de Pernambuco (UFPE), Recife, PE, Brasil; 5 Laboratório de Computação Científica e Análise Numérica, Universidade Federal de Alagoas (UFAL), Maceió, AL, Brazil; Tianjin University, CHINA

## Abstract

We present a new approach for handwritten signature classification and verification based on descriptors stemming from time causal information theory. The proposal uses the Shannon entropy, the statistical complexity, and the Fisher information evaluated over the Bandt and Pompe symbolization of the horizontal and vertical coordinates of signatures. These six features are easy and fast to compute, and they are the input to an One-Class Support Vector Machine classifier. The results are better than state-of-the-art online techniques that employ higher-dimensional feature spaces which often require specialized software and hardware. We assess the consistency of our proposal with respect to the size of the training sample, and we also use it to classify the signatures into meaningful groups.

## Introduction

The word *biometrics* is associated to human traits or behaviors which can be measured and used for individual recognition. In fact, the biometry recognition, as a personal authentication signal processing, can be used in situations or instances where users need to be security identified [1]. These kind of systems can either verify or identify.

Two types of biometrics can be defined according to the personal traits considered: *a)* *physical/physiological* which take into account the biological traits of users, like fingerprints, iris, face, hand, etc. *b)* *behavioral*, those which consider dynamic traits such as, voice, handwritten evidence and particular expressions. Biometric systems are attractive because of the enhanced security [1] provided by two main facts: (i) users do not have to remember passwords or carry access keys, (ii) it is difficult to steal, imitate or generate genuine biometric data.

The way we sign has the widest social and legal acceptance among pure behavioral biometric traits [2–6]. People sign every day to verify their identity, as this does not require any invasive measurement. Allegedly, this identification and identity verification modality is the most attacked.

Signatures are written by moving a pen over a surface, e.g., paper or a digitizing device. Handwritten signature verification is a problem in which the input signature (a test signature) is classified as genuine or forged. Although signatures are intended to serve as identity verification, the same person’s signature varies due to a number of factors and conditions.

Hilton [7] found that signatures have three main attributes: form, movement, and variation; movement being the most important. The author found that little variations occur over time once a signature style has been adopted. The signing processes can be described at high level as how the the brain recovers information from long term memory in which parameters such as size, shape, timing, etc., are specified, without any particular attention to detail. Genuine signatures are associated to a spurt of neural activity, whereas the forgery signatures are the result of deliberate handwriting which is characterized by a conscious attempt to reproduce [8, 9].

Two opposite mechanisms describing the signing process can be found in the literature. Longstaff and Heath [10] found evidence of chaotic behavior on the underlying dynamics of time series related to velocity profiles of handwritten texts. In opposition, most of the research in the field of signal verification considers the input information as well described by a random process, e.g. Hidden Markov Models [2–6]. Then, the dynamic input information acquired through a time sampling procedure must be considered as a discrete time random sequence.

Offline signature verification is based solely on the signature image, while online procedures require additional information. Our procedure exploits only the temporal information present in the signature coordinates and, thus, can be termed *quasi-offline*.

Following [2–6], we describe the three main stages of our work:

- **Data acquisition and pre-processing.** We perform *quasi-offline* recognition, as we only employ information about coordinates and do not require pressure, speed or pen-up movements data.
- **Feature extraction.** We tackle the problem with *parameter features*: signatures are characterized as a six-dimensional vector extracted from the original data.
- **Classification.** Our approach is related to *distance-based classifiers*, as we will make decisions based on the similarity of the features extracted from the test signature to a description of an ensemble of genuine signatures.

Our proposal relies on the use of time causal quantifiers based on information theory for the characterization of quasi-offline handwritten signatures: *normalized permutation Shannon entropy*, *permutation statistical complexity* and *permutation Fisher information measure.* These quantifiers have proved to be useful in the identification of chaotic and stochastic dynamics throughout the associated time series [11, 12]. Details and further references are provided in the Supplementary Information S1 File. Their evaluation is simple and fast, making them apt for the signature verification problem. We apply our proposal to the well-know MCYT online signature data base [13], but we only use time causal information about their trajectories.

We refer to “time causal information” to attest that the only causal information we use comes from the time ordering of the data. Mutual Information, Conditional Entropy, Transfer Entropy and other similar measures are excellent for identifying and quantifying relationships between processes, e.g. synchronization, causality, etc. [14]. This is not the case in our study, as we do not employ any other process apart from the observed coordinates along time. Those information theory measures would be of great value if we had data about, for instance, the neural activity that leads to the signatures, but we do not.

Our proposal consists, thus, in using features extracted from a nonparametric transformation of two time series. Other recent techniques have been proposed for the analysis of time series as, for instance, transforming them into complex networks [15–19], and using multiscale analysis [20]. These, and other similar approaches, produce excellent results at the price of heavy computational overload.

**Fig 1:**
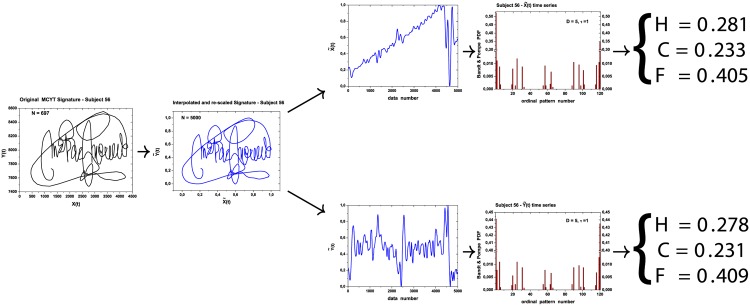
Diagram of the proposed procedure: original signature, interpolation, X and Y coordinates as time series, Band & Pompe histograms, entropy, statistical complexity and Fisher information.

Fig 1 sketches the complete workflow of our proposal. Signatures are the input; they are first scaled to fit an unitary square, and interpolated in order to have same number of data for all subjects. Then, the time series of both horizontal and vertical writing processes are extracted. These time series are then represented in a nonparametric manner using a time causal descriptor: the Bandt and Pompe symbolization [21]. A histogram of these symbols is then built for each coordinate, and information theory quantifiers are computed from these histograms: normalized Shannon entropy, Fisher’s information measure, and statistical complexity. After an exploratory data analysis, we show that simple dendrograms based on these quantifiers reveal meaningful groups of signatures. The signature stability of each of these groups is also evaluated. Finally, we propose using a One-Class Support Vector Machine for signature verification, and we show that this approach has better performance than state-of-the-art classifiers defined in feature spaces ten times larger than ours. With this, our proposal attains better results in less computational time for an application that, besides being relevant, requires fast responses.

Next section describes the database used in this study. In addition to the usual data flow, we present an exploratory data analysis (EDA) of the features that enhances their appropriateness for this problem. The expressiveness and usefulness of these descriptors for the problem of signature classification and verification follows in the sequence: we experiment their application to the test-bed.

## Handwritten signatures database

The present study is carried out on the freely available and widely used handwritten signatures database MCYT. In the following paragraph, we reproduce the main protocol and methodological details of the MCYT data base acquisition published by Ortega-Garcia and coworkers in [13, 22, 23].

“The acquisition of each on-line signature is accomplished dynamically using a graphics tablet. The signatures are acquired on a WACOM^©^ graphic tablet, model INTUOS A6 USB. The tablet resolution is 2540 lines/in (100 lines/mm), and the precision is ±0.25 mm. The maximum detection height is 10 mm (so also pen-up movements are considered), and the capture area is 127 mm (width) × 97 mm (height). This tablet provides the following discrete-time sequences: *a)* position *x*_*t*_ in the *x*-axis, *b)* position *y*_*t*_ in the *y*-axis, and *c)* also the time series corresponding to the pressure *p*_*t*_ applied by the pen, as well as the azimuth *γ*_*t*_ and altitude *φ*_*t*_ angles of the pen with respect to the tablet, not used in the present work. The sampling frequency is set to 100 Hz. Taking into account the Nyquist sampling criterion and that the maximum frequencies of the related biomechanical sequences are always under 20-30 Hz [24], this sampling frequency leads to a precise discrete-time signature representation. The signature corpus comprises genuine and shape-based highly skilled forgeries with natural dynamics [13, 23]. The forgeries are produced requesting each contributor to imitate other signers by writing naturally. For this task, they were given the printed signature to imitate and were asked not only to imitate the shape, but also to generate the imitation without artifacts such as breaks or slow-downs. Each signer contributes with 25 genuine signatures in five groups of five signatures each, and is forged 25 times by five different imitators. Since signers are concentrated in a different writing task between genuine signature sets, the variability between client signatures from different acquisition sets is expected to be higher than the variability of signatures within the same set. The total number of contributors in the MCYT is 330, and the total number of signatures present in the signature database is 16,500, half of them genuine signatures and the rest forgeries.”

We used the MCYT-100 subset of the database, which includes 100 subjects and for each one, 25 genuine and 25 skilled forged signatures. The only data we use are the *x*- and *y*-coordinates time series.

**Fig 2:**
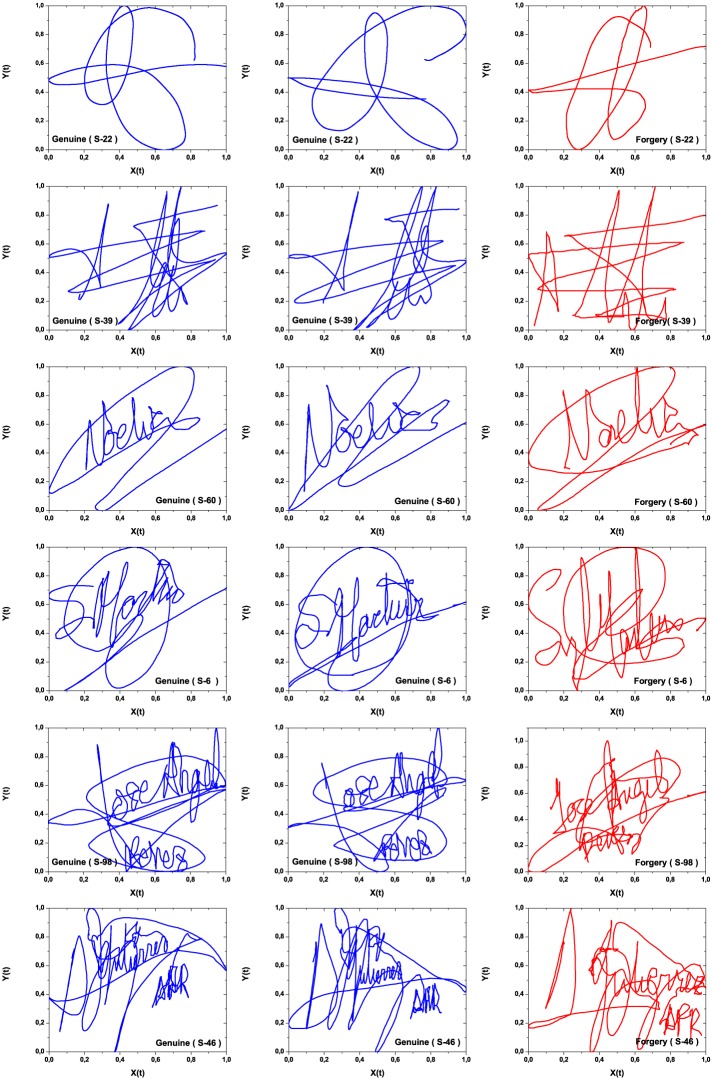
Six different subjects signatures from the MCYT database. Two genuine signatures (left, blue) and a skilled forgery (right, red). The two first signatures were classified as H1A and H1B, the following two to types H2A and H2B, and the last two to types H3A and H3B; cf. Sec. Signature classification.

Fig 2 presents examples of six subjects, being the first two columns genuine and the third column forgery signatures. In particular, one must note that the time series’ lengths are quite variable. We pre-processed each time series as follows: *a)* the coordinates were re-scaled into the unit square [0, 1] × [0, 1]; *b)* the original total number of data for each time series is expanded to *M* = 5000 points using a cubic Hermite polynomial. In this way, for each subject *k* (*k* = 1, …, 100) and associated signatures *j* (*j* = 1, …, 25) we will analyze two time series, denoted by $X_{j}^{\left(k;\alpha \right)}=\left\{0\leq {\tilde{x}}_{j;i}^{\left(k;\alpha \right)}\leq 1,i=1,\ldots ,M\right\}$ and $Y_{j}^{\left(k;\alpha \right)}=\left\{0\leq {\tilde{y}}_{j;i}^{\left(k;\alpha \right)}\leq 1,i=1,\ldots ,M\right\}$, in which the supra-index *α* = *G*, *F* denotes genuine and forgery signature, and $\tilde{x}$ and $\tilde{y}$ are the interpolated values, respectively.

## Signature features and exploratory data analysis

Handwritten classification and verification is an important and challenging problem due to two main factors. First, intra-personal variation in speed, pressure and inclination can be large, as signature consistency is often poor. Second, we can only obtain few samples from one person and no forgeries in practice. The reliability of extracted features is, thus, difficult to assess.

Developing an efficient and effective system for data acquisition is a challenging task. The volume of their databases grows boundlessly and soon becomes unmanageable, so reducing the raw data to parsimonious forms, without loosing important information, is at the core of intelligent solutions. We aim at discovering relevant low-dimensional features that, albeit promoting the reduction of data, are able to differentiate forgery from authentic signatures.

In this work we employ time causal information theory quantifiers; see details in the Supplementary Information S1 File. For each of the *k* subjects (*k* = 1, …, 100) in the database and its *j* associated signatures (25 genuine and 25 skilled forgery), two time series $X_{j}^{\left(k;\alpha \right)}$ and $Y_{j}^{\left(k;\alpha \right)}$ are extracted and transformed into Bandt and Pompe’s PDFs with pattern length (embedding dimension) *D* = 5 and time lag *τ* = 1 [21].

We denoted these PDFs as:

$$\begin{array}{ccc} P_{X;j}^{\left(k;\alpha \right)} & = & \;\text{Bandt}\;\text{and}\;\text{Pompe}’\text{s}\;\text{PDF}\;\text{of}\;{X_{j}^{\left(k;\alpha \right)}|}_{D,\tau },\;\text{and}\\ P_{Y;j}^{\left(k;\alpha \right)} & = & \;\text{Bandt}\;\text{and}\;\text{Pompe}’\text{s}\;\text{PDF}\;\text{of}\;{Y_{j}^{\left(k;\alpha \right)}|}_{D,\tau }, \end{array}$$

in which *j* = 1, …, 25, and *α* = *G*, *F* identify genuine and skilled forgery signatures, respectively.

We chose *D* = 5 after trying other values: *D* = 3, 4 led to too coarse histograms (not enough bins), while *D* = 6 (that requires counting 720 cases) produced too many zero-count bins. Note that the condition *M* ≫ *D*! is satisfied with *D* = 5. We used unlagged data (*τ* = 1) after checking that there were not significant changes with lagged *τ* = 2, 3 series.

We computed the normalized permutation Shannon entropy H, the permutation statistical complexity C, and the permutation Fisher information measure F from these PDFs, and the obtained values are denoted as:

$$\begin{array}{cccccc} H_{X;j}^{\left(k;\alpha \right)} & = & H[P_{X;j}^{\left(k;\alpha \right)}], & H_{Y;j}^{\left(k;\alpha \right)} & = & H[P_{Y;j}^{\left(k;\alpha \right)}];\\ C_{X;j}^{\left(k;\alpha \right)} & = & C[P_{X;j}^{\left(k;\alpha \right)}], & C_{Y;j}^{\left(k;\alpha \right)} & = & C[P_{Y;j}^{\left(k;\alpha \right)}];\\ F_{X;j}^{\left(k;\alpha \right)} & = & F[P_{X;j}^{\left(k;\alpha \right)}], & F_{Y;j}^{\left(k;\alpha \right)} & = & F[P_{Y;j}^{\left(k;\alpha \right)}]. \end{array}$$

We performed Exploratory Data Analysis (EDA) on these information theory quantifiers looking for simple descriptions of the data. We also used the Pearson correlation to measure the association between features. This analysis was performed using the R language and platform version 3.2.1 (http://www.R-project.org).

**Fig 3:**
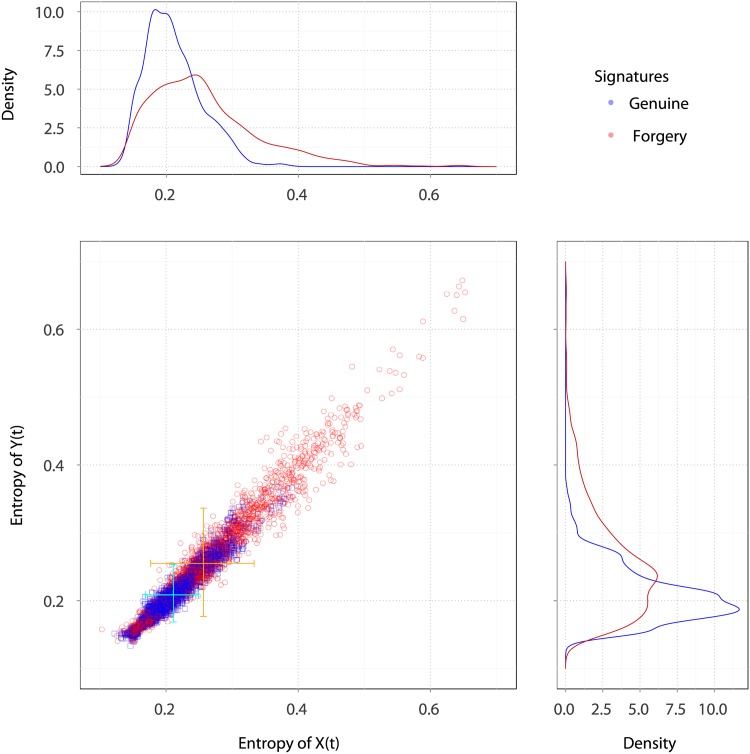
Scatter plot with marginal kernel density estimates of entropy quantifiers in both trajectory coordinates time series X and Y. Genuine (blue) and skilled forgery signatures (red points), 100 subjects. Marginal kernel densities depict the distribution of entropy quantifiers along both axes.

Fig 3 shows a scatterplot of the entropy for both the genuine and skilled forgery signatures. The 5000 points correspond to 25 genuine signatures (in blue) and 25 forgery signatures (in red) for each of the 100 subjects. Both types of signatures show similar association (Correlation): $\text{Corr}(H_{X;j}^{\left(k;G\right)},H_{Y;j}^{\left(k;G\right)})=0.9665$ and $\text{Corr}(H_{X;j}^{\left(k;F\right)},H_{Y;j}^{\left(k;F\right)})=0.9770$. The entropies of both types of signatures are overlapped and scattered elliptically. However, the bivariate mean and dispersion values differ.

Entropies are less dispersed in the genuine than in the skilled forgery signatures, a signal of the separability between them. Marginal density plots show the distribution of entropy for each coordinate of both types of signatures. These plots, in spite of being limited due to its marginal nature, reveal several modes, and suggest different dispersion patterns.

**Fig 4:**
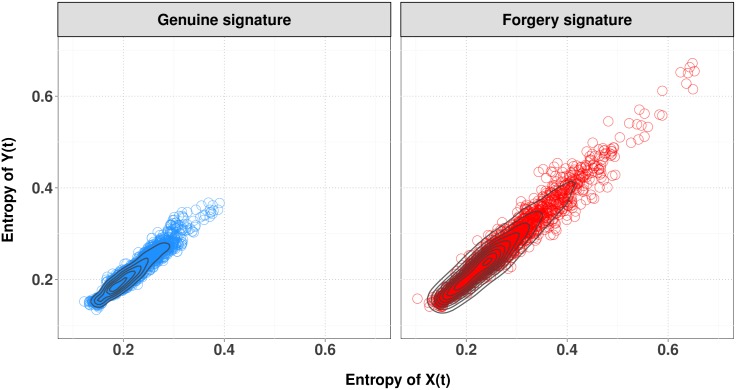
Contour plot superimposed on the scatterplot of entropy quantifiers for genuine (right panel) and skilled forgery signatures (left panel).

Fig 4 shows the contour plots of bivariate kernel density estimates for the entropy in genuine and forgery signatures. A number of features are immediately noticeable. The dispersion in the former group is much smaller than in the latter (less than 0.4). The kernel density estimates reveal skewness and a mild multimodality in the joint distribution of the data. Quite many points that are far from these curves and cluster centers. These points correspond to abnormal local estimates obtained in heterogeneous blocks, possibly induced by the presence of clusters. The modes in genuine signatures are smaller than in forgery signatures, and this may be used as discriminatory measure. Similar results are obtained for the Complexity and the Fisher information; these are reported in the Supplementary Information, see Figs A, B, C and D in S3 File, respectively.

## Signature classification

As pointed out by Boulétreal *et al.* [25], a signature is characterized by two aspects: *a)* a conscious one associated to the pattern signature; and *b)* an unconscious one which leads spontaneous movements constituting the drawing. These two factors produce high variability, being the amount of signature variability strongly writer-dependent. In fact, the signature *variability* or, conversely, the signature *stability* can be considered an important indicator for writer characterization [26]. Houmani and Garcia-Salicetti [26] argue that signature stability is required in genuine signatures to characterize a writer: signature variability reduces the ability to identify forgery. Also, complex enough signatures are required to guarantee a certain level of security, in the sense that the more complex a signature is, the more difficult it will be to forge it [26].

Boulétreal and collaborators [25, 27] propose a signature complexity measure related to signature legibility and based on fractal dimension. They classify writer styles into: highly cursive, very legible, separated, badly formed, and small writings, using only genuine signatures. Unfortunately, such resulting categories were not confronted to classifiers for performance analysis.

**Fig 5:**
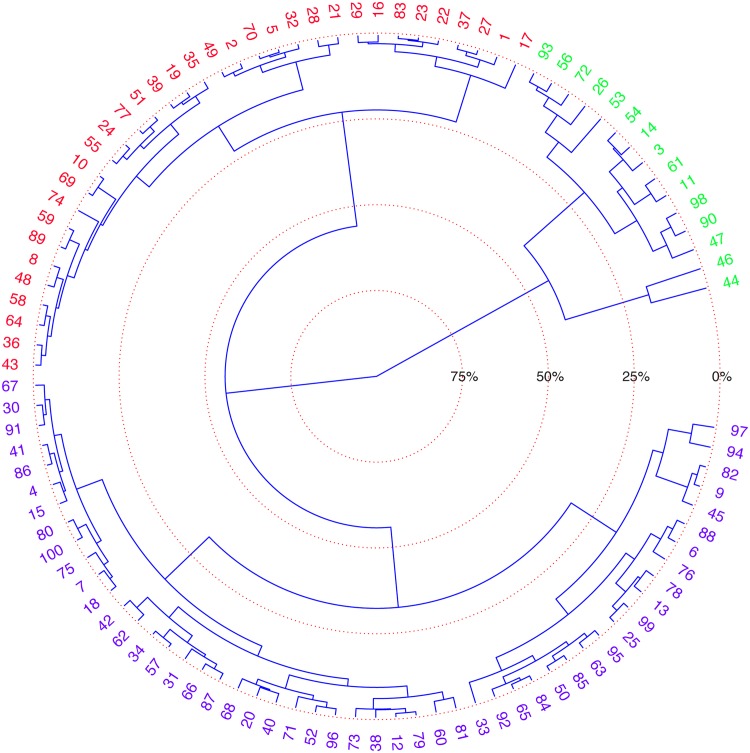
Neighbor-joining, rooted, circular dendrogram clustering of genuine signatures by entropy: H1, H2, and H3, in red, blue, and green, respectively.

We classified the one hundred genuine signatures in the MCYT-100 data base with causal information theory quantifiers: Normalized permutation Shannon entropy, permutation statistical complexity and permutation Fisher information measure of both **X** and **Y** trajectories. The mean and standard deviation values were clustered using the neighbor-joining method and an automatic Hierarchical Clustering with the Euclidean distance-based dissimilarity matrix. Each feature was treated independently, and the results are shown as circular dendrograms. Fig 5 shows the results of clustering the entropy. With this, we distinguish three classes of genuine signatures denoted by H1, H2, and H3.

The H1 group is the first group to form, i.e., the one comprised of the most similar individuals. It is formed below the 25% level, and it is composed by two subgroups: H1A and H1B. The H1A group is formed exclusively by oversimplified signatures made by mere loops without identifiable letters. It encompasses the following subjects: 1, 16, 17, 22, 23, 27, 29, 37, 83. The same group is formed when the other features are used. The H1B group is comprised of the following subjects: 2, 5, 8, 10, 19, 21, 24, 28, 32, 35, 36, 39, 43, 48, 49, 51, 55, 58, 59, 64, 69, 70, 74, 77, 89. Although these are simplified signatures, traces of letters and/or more complex curves appear and differentiate them from the members of H1A.

The H2 group is formed approximately at the 32% level, and, again, it is comprised of two distinct groups: H2A and H2B. The subjects that make the H2A group are: 4, 7, 12, 15, 18, 20, 30, 31, 34, 38, 40, 41, 42, 52, 57, 60, 62, 66, 67, 68, 71, 73, 75, 79, 80, 81, 86, 87, 91, 96, 100. It is composed by signatures with traces that resemble letters, but that are not perfectly identifiable, and that include circling traces of large or moderate size. Signatures in this group are kind of framed by large loops. The H2B group is similar to the previous one, i.e., it is formed by signatures with large and medium size circling traces, but with more identifiable letters than in the previous groups. Names and surnames are more readable in this group than in previous ones. It is formed by the following signatures: 6, 9, 13, 25, 33, 45, 50, 63, 65, 76, 78, 82, 84, 85, 88, 92, 94, 95, 97, 99.

The H3 group is formed at, approximately, the 43% level by the fusion of two other highly unbalanced subgroups: one, H3A, with only two subjects (44, 46) and the other, H3B, with thirteen subjects (3, 11, 14, 26, 47, 53, 54, 56, 61, 72, 90, 93, 98). These two clusters form at approximately the same level. The former is composed of calligraphic signatures where vertical traces predominate over horizontal ones. The latter is composed of highly cursive signatures, with separation between the surname and the family name.

The same results of clustering was obtained with the Manhattan (norm $L_{1}$) and Maximum distances ($L_{\infty }$ norm), showing that entropy is an expressive and stable quantifier. Similar analyses were carried with the permutation statistical complexity and permutation Fisher information (presented in Supplementary Information Figs A and B in S4 File). Complexity produces the same clusters identified by entropy, so it adds no new information. The Fisher information measure forms the same H1A group that was identified by the entropy, but with less cohesion, at about 15%. In other words, these nine subjects are more similar locally than globally. As with entropy, three main groups form at similar levels. The members of these clusters are slight variations of those identified using entropy, with very similar structure.

Table 1 presents the mean and standard deviation of the three quantifiers over the 25 genuine and 25 skilled forgery signatures (**X** and **Y** time series) for each of the typical subjects, split in types H1, H2, and H3. These data reveal interesting tendencies. Genuine signatures present quantifiers values lower than those corresponding to forgery signatures, and the latter also exhibit larger standard deviation. This may be explained by the imitative character of these signatures, however it deserves closer studies.

**Table 1:** Sample mean and standard deviation (S.D.) of the time series quantifiers for the 25 genuine (G) and 25 skilled forged (F) signatures, for each of the typical subjects: H1A, H1B, H2A, H2B, H3A, and H3B (same order as in Fig 2).

					Entropy		Complexity		Fisher Information	
*Type*	*Sub–Type*	Subject	Coordinate	Class	Mean	S.D.	Mean	S.D.	Mean	S.D.
H1	H1A	22	**X**	F	0.1568	0.0052	0.1490	0.0039	0.4688	0.0070
				G	0.1519	0.0019	0.1457	0.0015	0.4766	0.0035
			**Y**	F	0.1595	0.0071	0.1511	0.0052	0.4665	0.0097
				G	0.1512	0.0042	0.1447	0.0037	0.4734	0.0046
	H1B	39	**X**	F	0.2212	0.0384	0.1941	0.0257	0.4286	0.0147
				G	0.1749	0.0037	0.1620	0.0028	0.4497	0.0029
			**Y**	F	0.2270	0.0449	0.1980	0.0296	0.4277	0.0153
				G	0.1776	0.0043	0.1644	0.0031	0.4491	0.0035
H2	H2A	60	**X**	F	0.2482	0.0593	0.2112	0.0365	0.4212	0.0107
				G	0.2010	0.0056	0.1803	0.0040	0.4331	0.0031
			**Y**	F	0.2442	0.0544	0.2090	0.0339	0.4219	0.0134
				G	0.2079	0.0043	0.1861	0.0030	0.4315	0.0024
	H2B	6	**X**	F	0.2621	0.0584	0.2194	0.0334	0.4143	0.0137
				G	0.2337	0.0149	0.2032	0.0095	0.4205	0.0066
			**Y**	F	0.2648	0.0538	0.2218	0.0304	0.4136	0.0134
				G	0.2314	0.0102	0.2018	0.0067	0.4211	0.0050
H3	H3A	98	**X**	F	0.3236	0.0646	0.2529	0.0320	0.3937	0.0208
				G	0.2707	0.0101	0.2268	0.0064	0.4106	0.0032
			**Y**	F	0.3204	0.0794	0.2497	0.0388	0.3970	0.0208
				G	0.2664	0.0124	0.2243	0.0077	0.4105	0.0034
	H3B	46	**X**	F	0.3514	0.0641	0.2691	0.0294	0.3940	0.0156
				G	0.3480	0.0282	0.2720	0.0156	0.4019	0.0047
			**Y**	F	0.3419	0.0681	0.2639	0.0323	0.3940	0.0163
				G	0.3270	0.0263	0.2599	0.0148	0.4008	0.0052

**Fig 6:**
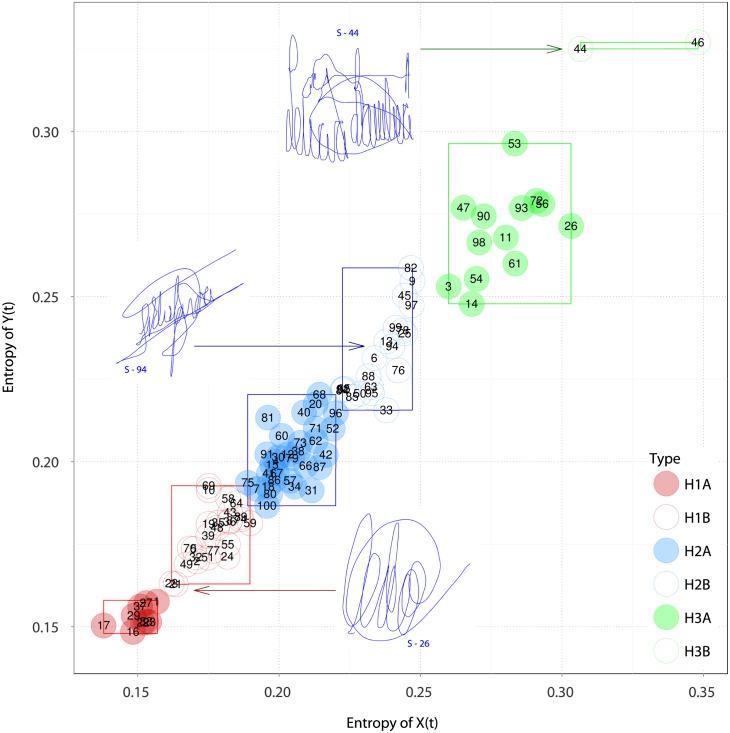
Classification by the rule of the parallelepiped of genuine signatures using entropy (one signature example from each of the three groups is shown). Each subject is identified by its ID.

The classification into subclasses of genuine signatures was also carried by the parallelepiped algorithm [28], arguably the simplest model-free classification procedure. Entropy leads to clusters with nice interpretability. Fig 6 shows the regions that define the three classes identified by the dendrogram based on entropy presented in Fig 5. All subclasses are well separated by disjoint boxes, except H1B and H2A that overlap slightly but without compromising the discrimination. The classes are preserved using this classification superimposed with Complexity and Fisher information features; see Figs C and D in S4 File.

## Signature stability measure

We now assess the stability of the features the classification procedure will use as input. Two measures of instability are computed over the PDFs obtained for each time series: one global (the Jensen-Shannon divergence [29–31]) and another local (the Jensen-Fisher divergence [32, 33]).

We propose using, for each subject, the square root of the Jensen-Shannon divergence over his/her 25 genuine signatures (denoted by *η*^(*k*)^) as a global index of instability

$$\begin{array}{c} \eta_{X}^{\left(k\right)}=\sqrt{S[\frac{1}{25}\sum_{j=1}^{25}P_{X;j}^{\left(k;G\right)}]-\frac{1}{25}\sum_{j=1}^{25}S[P_{X;j}^{\left(k;G\right)}]}, \end{array}$$ (1)

$$\begin{array}{c} \eta_{Y}^{\left(k\right)}=\sqrt{S[\frac{1}{25}\sum_{j=1}^{25}P_{Y;j}^{\left(k;G\right)}]-\frac{1}{25}\sum_{j=1}^{25}S[P_{Y;j}^{\left(k;G\right)}]}, \end{array}$$ (2)

in which, S[•] represents the Shannon entropy, $P_{X;j}^{\left(k;G\right)}$ and $P_{Y;j}^{\left(k;G\right)}$ are the Bandt-Pompe’s PDF associated to time series of coordinates $\tilde{x}$ and $\tilde{y}$ of the *j* genuine signature (*α* = *G*, *j* = 1, …, 25) of subject *k* (*k* = 1, …, 100).

Analogously, we define a local instability index using the Fisher information measure, F[•], and evaluating the Jensen-Fisher divergence. We then have

$$\begin{array}{c} \xi_{X}^{\left(k\right)}=\sqrt{\frac{1}{25}\sum_{j=1}^{25}F[P_{X;j}^{\left(k;G\right)}]-F[\frac{1}{25}\sum_{j=1}^{25}P_{X;j}^{\left(k;G\right)}]}, \end{array}$$ (3)

$$\begin{array}{c} \xi_{Y}^{\left(k\right)}=\sqrt{\frac{1}{25}\sum_{j=1}^{25}F[P_{Y;j}^{\left(k;G\right)}]-F[\frac{1}{25}\sum_{j=1}^{25}P_{Y;j}^{\left(k;G\right)}]}. \end{array}$$ (4)

**Fig 7:**
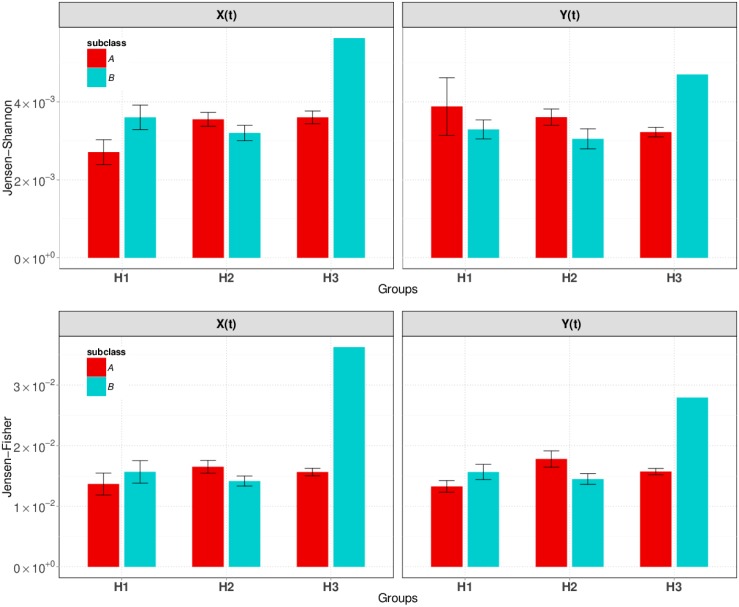
Global Jensen-Shannon (top) and local Jensen-Fisher (bottom) measures of instability in genuine signatures. Bars show the mean, and lines show the standard error over the subjects. The standard error of H3B is not plotted because there are only two subjects in this class.

Fig 7 shows the plots of mean with standard error bars of instability index calculated by each type of genuine signatures by subclasses as obtained from preclasification. The first observation is that the Jensen-Fisher local measure of instability (bottom) is the same in the horizontal (left) and right (left) time series, whereas it changes when measured by the Jensen-Shannon global measure (top).

The global measure of instability indicates that the most unstable group of genuine signatures is H3B, but only two samples are available in this class. Both H2 classes exhibit similar instabilities in both horizontal and vertical time series **X** and **Y**. The **X** and **Y** time series show a symmetrical behavior in class H1: **X** is more stable than **Y** in H1A, whereas **Y** is more stable than **X** in H1B. The least variable instability is observed in the H2 class.

All mean local stabilities, except that of H3B, are similar in the horizontal and vertical directions. The subclass H3B is, again, the most unstable, but it is more stable in the vertical direction.

Overall, the measured instability is small in all subclasses granting, thus, stable classification results based on these features.

## Quasi-offline signature verification

The problem we have at hand consists of identifying suspicious signatures given that we only have examples from genuine signatures. In practice, it is too expensive, too hard or even impossible to obtain a significant number of good quality forgery signatures for every possible individual in the data base. This, thus, configures a One-Class classification problem.

Support Vector Machines (SVMs) are suitable for solving machine learning problems even in large dimensional feature spaces [34–36]. We provide a brief description of SVMs and One-Class SVMs in the Supplementary Information S2 File along with a toy example with simulated data. We used the libsvm (version 2.0) tool, linked with the R software that implements SVM classification and regression, and One-Class SVMs (OC-SVM) [37] tools, with the default parameters.

We assess the consistency of our procedure in a reproducible manner by evaluating the performance of the proposed verification system for different training samples. Were selected random samples of size *n* = 5, 10, 14, 18, 22 of genuine signatures for each user. Table 2 presents the average value of all performance metrics using *σ*^2^ = 10 (see Supplementary Information S2 File). The observed Accuracy (ACC) suggests that the larger the training sample is the better the performance is. The Area Under the ROC Curve (AUC) presents a similar tendency, and its average is larger than 0.88, indicating that our verification system produces excellent classification.

**Table 2:** Performance of the system trained with varying number *n* of samples of genuine signatures; ↑ and ↓ denote measures of quality (the higher the better) and of error (the smaller the better), respectively.

*n*	ACC (↑)	AUC (↑)	EER(%) (↓)
5	0.6940	0.8816	0.1890
10	0.7678	0.8940	0.1711
14	0.8144	0.8975	0.1634
18	0.8250	0.8866	0.1731
22	0.8389	0.8909	0.1632

As mentioned in the introduction, the two methodologies with best results are those based on Dynamic Time Warping (DTW) and Hidden Markov Models (HMM). In the following we compare our proposal with these two recent state-of-the-art methods using the Equal Error Rate, EER(%) over the same data base:

- Fierrez-Aguilar *et al.* [38], ERR(%) = 2.12 (five training signatures; Global (Parzen WC) and local (HMM) experts function);
- Fierrez-Aguilar *et al.* [22], ERR(%) = 0.74 (ten training signatures; HMM based algorithm);
- Pascual-Gaspar *et al.* [39], ERR(%) = 1.23 (five training signatures; DTW-bases algorithm, result with scenario-dependent optimal features.

The results of our proposal using five (ten, respectively) training samples, are ERR(%) = 0.19 (0.17, respectively). Our system, thus, provides better performance using similar number of training signatures (see Table 2 for more details).

In the following we analyze the performance of the proposed procedure applied selectively to the pre-classified samples. Table 3 presents the performance of the system when applied to genuine pre-classified signatures. For all classes, larger training samples lead to larger average ACC. The best average AUC are observed for the class H2, followed by H1 and H3. This indicates that H2 signatures are easily identifiable. Note that the mean values of ERR(%) for H2 are smaller than H1 and H3. The ERR(%) values in H3 indicate that identifying forgeries in this class is hard.

**Table 3:** Performance of the classification of pre-classified samples varying the number *n* of samples of genuine signatures used for training; same coding as in Table 2.

Class	*n*	ACC (↑)	AUC (↑)	EER(%) (↓)
H1	5	0.6758	0.8692	0.1976
	10	0.7566	0.8828	0.1812
	14	0.8039	0.8857	0.1717
	18	0.8217	0.8894	0.1662
	22	0.8277	0.8788	0.1631
H2	5	0.7059	0.8945	0.1784
	10	0.7819	0.9079	0.1548
	14	0.8284	0.9096	0.1509
	18	0.8327	0.8900	0.1734
	22	0.8515	0.8996	0.1608
H3	5	0.6948	0.8653	0.2053
	10	0.7450	0.8720	0.2036
	14	0.7907	0.8832	0.1874
	18	0.8062	0.8686	0.1874
	22	0.8214	0.8889	0.1716

## Conclusions

We proposed a quasi-offline procedure for identifying skilled forgery of handwritten signatures using time causal information Theory quantifiers and One-Class Support Vector Machines. This is a competitive proposal from the computational viewpoint as it uses only the signatures coordinates, and it produces better results than state-of-the-art techniques. The improvement is obtained in a six-dimensional feature space, while other techniques employ forty or more features. As a consequence, the processing time, memory and storage required are reduced and, at the same time, the procedure is less prone to the problems induced by the curse of dimensionality. Such improvements make our proposal apt for becoming stand-alone application in, e.g., mobile banking.

The technique also produces meaningful classification of the input data, as it is able to separate different types of signatures. To the best of our knowledge, this is the first time information theory quantifiers have been used for this problem.

The central contribution is the use of the Bandt and Pompe (BP) PDF symbolization which is invariant to a number of transformations of the input data. In fact, the original time series are pre-processed only to facilitate the signal sampling, and this scaling has no effect on the BP PDFs. This representation, which is sensitive to the time causality, is able to capture essential dynamical characteristics of the signatures that lead to excellent discrimination between skilled forgery and genuine handwritten signatures, despite the high variability the data possess. Additionally, obtaining the BP PDFs is computationally simple and efficient.

Only six information theory features are required for the classification, three from each horizontal and vertical direction: Shannon entropy, statistical complexity, and Fisher information. This contrasts many state-of-the-art works that require features in high-dimensional spaces, e.g. forty or even more. As said, our proposal does not require highly specialized hardware able to capture signature speed, pressure, orientation, etc.

The classification was performed by a One-Class Support Vector Machine trained with genuine signatures. The learned rule is consistent with respect to the number of training samples, and with as few as five examples it surpasses the performance of recent successful techniques. We assessed the performance of our proposal using the same data base employed in the current literature, with also the same measures of quality and error.

Future work includes the use of other variables already available in the MCYT data base (pressure, and azimuth and altitude angles), along with other features, e.g. clustering coefficient entropy, network clustering coefficient, permutation min-entropy [40–42], and clustering and classification techniques as, for instance, deep learning [43].

## Supporting Information

S1 File Supporting Information file that contains additional material about Information Theory Quantifiers. (PDF)

S2 File Supporting Information file that contains additional material about Support Vector Machines and One-Class Support Vector Machines. (PDF)

S3 File Supporting Information file that contains additional material about Exploratory Data Analysis. (PDF)

S4 File Supporting Information file that contains additional material about Signature Classification. (PDF)
