# Supplementary material for: Classification and Verification of Handwritten Signatures with Time Causal Information Theory Quantifiers
Source: PLoS One. 2016 Dec 1;11(12):e0166868. doi: 10.1371/journal.pone.0166868 (PMC5131934; doi:10.1371/journal.pone.0166868)
Supplement: S1 File — (PDF) [file pone.0166868.s001.pdf]

Information Theory quantifiers

Physics, as well as, other scientific disciplines like biology or finance, can be considered observational sciences, that is, they try to infer properties of an unfamiliar system from the analysis of measured time record of it behavior (time series). Dynamical systems are systems that evolve in time. In practice, one may only be able to measure a scalar time series $\mathcal{X}(t)$ which may be a function of variables $\mathcal{V} = \{v_1, v_2, \cdots, v_k\}$ describing the underlying dynamics (i.e. $d\mathcal{V}/dt = f(\mathcal{V})$. Then, the natural question is, from $\mathcal{X}(t)$ how much we can learn about the dynamics of the system. In a more formal way, given a system, be it natural or man-made, and given an observable of such system whose evolution can be tracked through time, a natural question arises: how much information is this observable encoding about the dynamics of the underlying system? The information content of a system is typically evaluated via a probability distribution function (PDF) P describing the apportionment of some measurable or observable quantity, generally a time series $\mathcal{X}(t)$. Quantifying the information content of a given observable is therefore largely tantamount to characterizing its probability distribution. This is often done with the wide family of measures called Information Theory quantifiers [1]. We can define Information Theory quantifiers as measures able to characterize relevant properties of the PDF associated with these time series, and in this way we should judiciously extract information on the dynamical system under study.

Shannon entropy, Fisher Information measure, and Statistical Complexity

Entropy is a basic quantity with multiple field-specific interpretations: for instance, it has been associated with disorder, state-space volume, and lack of information [2]. When dealing with information content, the Shannon entropy is often considered as the foundational and most natural one [3, 4].

Entropy, regarded as a measure of uncertainty, is the most paradigmatic example of these information quantifiers. Given a continuous probability distribution function (PDF) $\rho(x)$ with $x \in \Omega \subset \mathbb{R}$ and $\int_\Omega \rho(x)\, dx = 1$, its associated *Shannon Entropy* S [3, 4] is defined by

$$\mathrm{S}[\rho] \;=\; -\int_\Omega \rho(x) \ln\left[\rho(x)\right] dx. \tag{S1.1}$$

It is a global measure, that is, it is not too sensitive to strong changes in the distribution taking place on a small-sized region of Ω. Such is not the case with *Fisher's Information Measure* (FIM) $\mathcal{F}$[5, 6], which constitutes a measure of the gradient content of the distribution ρ, thus being quite sensitive even to tiny localized perturbations. It reads

$$\mathcal{F}[\rho] \;=\; \int \frac{|\vec{\nabla}\rho(x)|^2}{\rho(x)}\, dx \;=\; 4 \int |\vec{\nabla}\psi(x)|^2\, dx, \quad \text{where } \psi(x) \;=\; \sqrt{\rho(x)}\;. \tag{S1.2}$$

The Fisher Information Measure can be variously interpreted as a measure of the ability to estimate a parameter, as the amount of information that can be extracted from a set of measurements, and also as a measure of the state of disorder of a system or phenomenon[6], its most important property being the so-called Cramer-Rao bound. It is important to remark that the gradient operator significantly influences the contribution of minute local ρ-variations to the Fisher information value, accordingly, this quantifier is called "local" [6]. Note that the Shannon entropy decreases with the distribution skewness, while the Fisher information increases.

Local sensitivity is useful in scenarios whose description necessitates an appeal to a notion of "order". In the previous definition of FIM (Eq. (S1.2)) the division by $\rho(x)$ is not convenient

if $\rho(x) \to 0$ at certain points of the support Ω. We avoid this if we work with real probability amplitudes, by means of the alternative expression that employs $\psi(x)$ [5, 6]. This form requires no divisions, and shows that $\mathcal{F}$ simply measures the gradient content in $\psi(x)$.

Let now $P = \{p_i; i = 1, \ldots, N\}$ with $\sum_{i=1}^{N} p_i = 1$, be a discrete probability distribution, with N the number of possible states of the system under study. The Shannon's logarithmic information measure reads

$$\mathrm{S}[P] \; = \; -\sum_{i=1}^{N} p_i \ln\left[p_i\right] \; . \tag{S1.3}$$

This can be regarded to as a measure of the uncertainty associated (information) to the physical process described by P. For instance, if $\mathrm{S}[P] = \mathrm{S}_{\min} = 0$, we are in position to predict with complete certainty which of the possible outcomes i, whose probabilities are given by p_i, will actually take place. Our knowledge of the underlying process described by the probability distribution is maximal in this instance. In contrast, our knowledge is minimal for a uniform distribution $P_e = \{p_i = 1/N, \forall i = 1, \ldots, N\}$ since every outcome exhibits the same probability of occurrence, and the uncertainty is maximal, i.e., $\mathrm{S}[P_e] = \mathrm{S}_{\max} = \ln N$. In the discrete case, we define a "normalized" Shannon entropy, $0 \leq \mathcal{H} \leq 1$, as

$$\mathcal{H}[P] \; = \; \mathrm{S}[P]/\mathrm{S}_{\max} \; . \tag{S1.4}$$

The concomitant problem of loss of information due to the discretization has been thoroughly studied (see, for instance, [7, 8] and references therein) and, in particular, it entails the loss of Fisher's shift-invariance, which is of no importance for our present purposes. For the FIM we take the expression in terms of real probability amplitudes as starting point, then a discrete normalized FIM, $0 \leq \mathcal{F} \leq 1$, convenient for our present purposes, is given by

$$\mathcal{F}[P] \; = \; F_0 \sum_{i=1}^{N-1} \left[\sqrt{p_{i+1}} - \sqrt{p_i}\right]^2 \; . \tag{S1.5}$$

It has been extensively discussed that this discretization is the best behaved in a discrete environment [9, 10]. Here the normalization constant F_0 reads

$$F_0 \; = \; \begin{cases} 1, & \text{if } p_{i^*} = 1 \text{ for } i^* = 1 \text{ or } i^* = N \text{ and } p_i = 0, \forall i \neq i^*, \\ 1/2, & \text{otherwise} \; . \end{cases} \tag{S1.6}$$

Complexity denotes a state of affairs that one can easily appreciate when confronted with it; however, is rather difficult to define it quantitatively, probably due to the fact that there is no universal definition of complexity. In between the two special instances of perfect order and complete randomness, a wide range of possible degrees of physical structure exists that should be reflected in the features of the underlying probability distribution P. One would like to assume that the degree of correlational structures would be adequately captured by some functional $\mathcal{C}[P]$ in the same way that Shannon's entropy $\mathrm{S}[P]$ [3] "captures" randomness.

Clearly, the ordinal structures present in a process is not quantified by randomness measures, and consequently, measures of statistical or structural complexity are necessary for a better understanding (characterization) of the system dynamics represented by their time series [11]. The opposite extremes of perfect order and maximal randomness are very simple to describe, because they do not have any structure. The complexity should be zero in these cases. At a given distance from these extremes, a wide range of possible ordinal structures exists. Complexity can be characterized by a certain degree of organization, structure, memory, regularity, symmetry, and patterns

[12]. The complexity measure does much more than satisfy the boundary conditions of vanishing in the high- and low-entropy limits. In particular the maximum complexity occurs in the region between the system's perfectly ordered state and the perfectly disordered one. Complexity is allows us to detect essential details of the dynamics, and more importantly to characterize the correlational structure of the orderings present in the time series.

The perfect crystal and the isolated ideal gas are two typical examples of systems with minimum and maximum entropy, respectively. However, they are also examples of simple models and therefore of systems with zero complexity, as the structure of the perfect crystal is completely described by minimal information (i.e., distances and symmetries that define the elementary cell) and the probability distribution for the accessible states is centered around a prevailing state of perfect symmetry. On the other hand, all the accessible states of the ideal gas occur with the same probability and can be described by a "simple" uniform distribution.

Statistical complexity is often characterized by the paradoxical situation of a complicated dynamics generated from relatively simple systems. Obviously, if the system itself is already involved enough and is constituted by many different parts, it clearly may support a rather intricate dynamics, but perhaps without the emergence of typical characteristic patterns [13]. Therefore, a complex system does not necessarily generate a complex output. Statistical complexity is therefore related to patterned structures hidden in the dynamics, emerging from a system which itself can be much simpler than the dynamics it generates [13].

According to López-Ruiz, Mancini and Calbet [14], and using an oxymoron, an object, a procedure, or system is said to be complex when it does not exhibit patterns regarded as simple. It follows that a suitable complexity measure should vanish both for completely ordered and for completely random systems and cannot only rely on the concept of information (which is maximal and minimal for the above mentioned systems). A suitable measure of complexity can be defined as the product of a measure of information and a measure of disequilibrium, i.e. some kind of distance from the equiprobable distribution of the accessible states of a system. In this respect, Rosso and coworkers [16] introduced an effective *Statistical Complexity Measure* (SCM) $\mathcal{C}$, that is able to detect essential details of the dynamical processes underlying the dataset.

Based on the seminal notion advanced by López-Ruiz *et al.* [14], this statistical complexity measure[15, 16] is defined through the functional product form

$$\mathcal{C}[P] \;=\; \mathcal{Q}_J[P,P_e]\cdot\mathcal{H}[P] \tag{S1.7}$$

of the normalized Shannon entropy $\mathcal{H}$, see Eq. (S1.4), and the disequilibrium $\mathcal{Q}_J$ defined in terms of the Jensen-Shannon divergence $\mathcal{J}[P,P_e]$. That is,

$$\mathcal{Q}_J[P,P_e] \;=\; Q_0\mathcal{J}[P,P_e] \;=\; Q_0\{\mathrm{S}[(P+P_e)/2]-\mathrm{S}[P]/2-\mathrm{S}[P_e]/2\}, \tag{S1.8}$$

the above-mentioned Jensen-Shannon divergence and Q_0, a normalization constant such that $0\le\mathcal{Q}_J\le 1$:

$$Q_0 \;=\; -2\left\{\frac{N+1}{N}\ln(N+1)-\ln(2N)+\ln N\right\}^{-1}\;, \tag{S1.9}$$

are equal to the inverse of the maximum possible value of $\mathcal{J}[P,P_e]$. This value is obtained when one of the components of P, say p_m, is equal to one and the remaining p_j are zero.

The Jensen-Shannon divergence, which quantifies the difference between probability distributions, is especially useful to compare the symbolic composition between different sequences[17, 18, 19]. Note that the above introduced SCM depends on two different probability distributions: one

associated with the system under analysis, P, and the other the uniform distribution, P_e. Furthermore, it was shown that for a given value of $\mathcal{H}$, the range of possible $\mathcal{C}$ values varies between a minimum $\mathcal{C}_{min}$ and a maximum $\mathcal{C}_{max}$, restricting the possible values of the SCM [20]. Thus, it is clear that important additional information related to the correlational structure between the components of the physical system is provided by evaluating the statistical complexity measure.

If our system, with associated discrete PDF, lies in a very ordered state, will be represented by an extremely narrow PDF, that is almost all the p_i–values are almost zero except for a particular state $k \neq i$ with $p_k \cong 1$,then both the normalized Shannon entropy and statistical complexity are close to zero ($\mathcal{H} \approx 0$ and $\mathcal{C} \approx 0$), and the normalized Fisher's information measure is close to one ($\mathcal{F} \approx 1$). On the other hand, when the system under study is represented by a very disordered state, that is when all the p_i–values oscillate around the same value, we have $\mathcal{H} \approx 1$ while $\mathcal{C} \approx 0$ and $\mathcal{F} \approx 0$. One can state that the general FIM–behavior of the present discrete version (Eq. (S1.5)), is opposite to that of the Shannon entropy, except for periodic motions. The local sensitivity of FIM for discrete–PDFs is reflected in the fact that the specific "$i-$ordering" of the discrete values p_i must be seriously taken into account in evaluating the sum in Eq. (S1.5). This point was extensively discussed by Rosso and co-workers [21, 22]. The summands can be regarded to as a kind of "distance" between two contiguous probabilities. Thus, a different ordering of the pertinent summands would lead to a different FIM-value, hereby its local nature. In the present work, we follow the Lehmer lexicographic order [23] in the generation of Bandt and Pompe PDF (see next section). Given the local character of FIM, when combined with a global quantifier as the normalized Shannon entropy, conforms the Shannon–Fisher plane, $\mathcal{H} \times \mathcal{F}$, introduced by Vignat and Bercher [24]. These authors showed that this plane is able to characterize the non-stationary behavior of a complex signal.

The Bandt and Pompe approach to the PDF determination

The evaluation of the Information Theory derived quantifiers, like those previously introduced (Shannon entropy, Fisher information and statistical complexity), suppose some prior knowledge about the system; specifically, a probability distribution associated to the time series under analysis should be provided beforehand. The determination of the most adequate PDF is a fundamental problem because the PDF P and the sample space Ω are inextricably linked.

Usual methodologies assign to each time point of the series $\mathcal{X}(t)$ a symbol from a finite alphabet $\mathfrak{A}$, thus creating a *symbolic sequence* that can be regarded to as a *non causal coarse grained* description of the time series under consideration. As a consequence, order relations and the time scales of the dynamics are lost. The usual histogram technique corresponds to this kind of assignment. *Causal information* may be duly incorporated if information about the past dynamics of the system is included in the symbolic sequence, i.e., symbols of alphabet $\mathfrak{A}$ are assigned to a portion of the phase-space or trajectory.

Many methods have been proposed for a proper selection of the probability space (Ω, P). Among others, of type non causal coarse grained, we can mention frequency counting [25], procedures based on amplitude statistics [26], binary symbolic dynamics [27], Fourier analysis [28], or wavelet transform [29]. The suitability of each of the proposed methodologies depends on the peculiarity of data, such as stationarity, length of the series, the variation of the parameters, the level of noise contamination, etc. In all these cases, global aspects of the dynamics can be somehow captured, but the different approaches are not equivalent in their ability to discern all relevant physical details. Bandt and Pompe (BP)[30] introduced a simple and robust symbolic methodology that takes into account time causality of the time series (causal coarse grained methodology)

by comparing neighboring values in a time series. The symbolic data are: *(i)* created by ranking the values of the series; and *(ii)* defined by reordering the embedded data in ascending order, which is tantamount to a phase space reconstruction with embedding dimension (pattern length) D and time lag τ. In this way, it is possible to quantify the diversity of the ordering symbols (patterns) derived from a scalar time series.

Note that the appropriate symbol sequence arises naturally from the time series, and no model-based assumptions are needed. In fact, the necessary "partitions" are devised by comparing the order of neighboring relative values rather than by apportioning amplitudes according to different levels. This technique, as opposed to most of those in current practice, takes into account the temporal structure of the time series generated by the physical process under study. As such, it allows us to uncover important details concerning the ordinal structure of the time series [31, 32, 33, 34] and can also yield information about temporal correlation [35, 36].

It is clear that this type of analysis of a time series entails losing details of the original series' amplitude information. Nevertheless, by just referring to the series' intrinsic structure, a meaningful difficulty reduction has indeed been achieved by BP with regard to the description of complex systems. The symbolic representation of time series by recourse to a comparison of consecutive ($\tau = 1$) or nonconsecutive ($\tau > 1$) values allows for an accurate empirical reconstruction of the underlying phase-space, even in the presence of weak (observational and dynamic) noise [30]. Furthermore, the ordinal patterns associated with the PDF are invariant with respect to nonlinear monotonous transformations. Accordingly, nonlinear drifts or scaling artificially introduced by a measurement device will not modify the estimation of quantifiers, a nice property if one deals with experimental data (see, e.g., [37]). These advantages make the BP methodology more convenient than conventional methods based on range partitioning, i.e., a PDF based on histograms.

To use the BP methodology[30] for evaluating the PDF, P, associated with the time series (dynamical system) under study, one starts by considering partitions of the D-dimensional space that will hopefully "reveal" relevant details of the ordinal structure of a given one-dimensional time series $\mathcal{X}(t) = \{x_t; t = 1, \ldots, M\}$ with embedding dimension $D > 1$ ($D \in \mathbb{N}$) and time lag τ ($\tau \in \mathbb{N}$). We are interested in "ordinal patterns" of order (length) D generated by

$$(s) \mapsto \left(x_{s-(D-1)\tau}, x_{s-(D-2)\tau}, \ldots, x_{s-\tau}, x_s\right) \ , \tag{S1.10}$$

which assign to each time s the D-dimensional vector of values at times $s-(D-1)\tau, \ldots, s-\tau, s$. Clearly, the greater D, the more information on the past is incorporated into our vectors. By "ordinal pattern" related to the time (s), we mean the permutation $\pi = (r_0, r_1, \ldots, r_{D-1})$ of $[0, 1, \ldots, D-1]$ defined by

$$x_{s-r_{D-1}\tau} \le \ x_{s-r_{D-2}\tau} \le \cdots \le \ x_{s-r_1\tau} \le x_{s-r_0\tau}. \tag{S1.11}$$

In this way the vector defined by Eq. (S1.10) is converted into a unique symbol π. We set $r_i < r_{i-1}$ if $x_{s-r_i} = x_{s-r_{i-1}}$ for uniqueness, although ties in samples from continuous distributions have null probability.

In order to illustrate BP method, we will consider a simple example: a time series with seven ($M = 7$) values $\mathcal{X} = \{4, 7, 9, 10, 6, 11, 3\}$ and we evaluate the BP-PDF for $D = 3$ and $\tau = 1$. In this case the state space is divided into 3! partitions and 6 mutually exclusive permutation symbols are considered. The triplet $(4, 7, 9)$ and $(7, 9, 10)$ represent the permutation pattern [012] since they are in increasing order. On the other hand, $(9, 10, 6)$ and $(6, 11, 3)$ correspond to the permutation pattern [201] since $x_{s+2} < x_s < x_{s+1}$, while $(10, 6, 11)$ has the permutation

pattern [102] with $x_{s+1} < x_s < x_{s+2}$. Then, the associated probabilities to the 6 patterns are: $p([012]) = p([201]) = 2/5$; $p([102]) = 1/5$; $p([021]) = p([120]) = p([210]) = 0$.

Fig A illustrates the construction principle of the ordinal patterns of length $D = 2$, 3 and 4 with $\tau = 1$[38]. Consider the sequence of observations $\{x_0, x_1, x_2, x_3\}$. For $D = 2$, there are only two possible directions from x_0 to x_1: up and down. For $D = 3$, starting from x_1 (up) the third part of the pattern can be above x_1, below x_0, or between x_0 and x_1. A similar situation can be found starting from x_1 (down). For $D = 4$, for each one of the six possible positions for x_2, there are four possible localizations for x_3, yielding $D! = 4! = 24$ different possible ordinal patterns. In Fig A, full circles and continuous lines represent the sequence values $x_0 < x_1 > x_2 > x_3$, which leads to the pattern $\pi = [0321]$. A graphical representation of all possible patterns corresponding to $D = 3, 4$ and 5 can be found in Fig 2 of Parlitz *et al.*[38].

For all the $D!$ possible orderings (permutations) π_i when embedding dimension is D, and time-lag τ, their relative frequencies can be naturally computed according to the number of times this particular order sequence is found in the time series, divided by the total number of sequences,

$$p(\pi_i) = \frac{\#\{s | s \leq N - (D-1)\tau; (s) \text{ is of type } \pi_i\}}{N - (D-1)\tau}, \tag{S1.12}$$

where # denotes cardinality. Thus, an ordinal pattern probability distribution $P = \{p(\pi_i), i = 1, \ldots, D!\}$ is obtained from the time series.

The embedding dimension D plays an important role in the evaluation of the appropriate probability distribution, because D determines the number of accessible states $D!$ and also conditions the minimum acceptable length $M \gg D!$ of the time series that one needs in order to work with reliable statistics [39]. In the present work, we follow the Lehmer lexicographic order [23] in the generation of Bandt and Pompe PDF.

Regarding the selection of the parameters, Bandt and Pompe suggested working with $4 \leq D \leq 6$, and specifically considered a time lag $\tau = 1$ in their cornerstone paper [30]. Nevertheless, it is clear that other values of τ could provide additional information. It has been recently shown that this parameter is strongly related, if it is relevant, to the intrinsic time scales of the system under analysis [40, 41, 42].

Additional advantages of the method reside in *i)* its simplicity (it requires few parameters: the pattern length/embedding dimension D and the time lag τ), and *ii)* the extremely fast nature of the calculation process. The BP methodology can be applied not only to time series representative of low dimensional dynamical systems, but also to any type of time series (regular, chaotic, noisy, or reality based). In fact, the existence of an attractor in the D-dimensional phase space in not assumed. The only condition for the applicability of the BP method is a very weak stationary assumption: for $k \leq D$, the probability for $x_t < x_{t+k}$ should not depend on t. For a review of BP's methodology and its applications to physics, biomedical and econophysics signals see Zanin *et al.* [43]. Moreover, Rosso *et al.* [31] show that the above mentioned quantifiers produce better descriptions of the process associated dynamics when the PDF is computed using BP rather than using the usual histogram methodology.

The BP proposal for associating probability distributions to time series (of an underlying symbolic nature) constitutes a significant advance in the study of nonlinear dynamical systems [30]. The method provides univocal prescription for ordinary, global entropic quantifiers of the Shannon-kind. However, as was shown by Rosso and coworkers [21, 22], ambiguities arise in applying the BP technique with reference to the permutation of ordinal patterns. This happens if one wishes to employ the BP-probability density to construct local entropic quantifiers, like the Fisher information measure, which would characterize time series generated by nonlinear

dynamical systems.

The local sensitivity of the Fisher information measure for discrete PDFs is reflected in the fact that the specific "i-ordering" of the discrete values p_i must be seriously taken into account in evaluating Eq. (S1.5). The numerator can be regarded to as a kind of "distance" between two contiguous probabilities. Thus, a different ordering of the summands will lead, in most cases, to a different Fisher information value. In fact, if we have a discrete PDF given by $P = \{p_i, i = 1, \dots, N\}$, we will have $N!$ possibilities for the i-ordering.

The question is, which is the arrangement that one could regard as the "proper" ordering? The answer is straightforward in some cases, like the one that pertains to histogram-based PDFs. For extracting a time-series PDF P via an histogram procedure, one first divides the interval $[a, b]$ (with a and b the minimum and maximum amplitude values in the time series) into a finite number N_{bin} ($N \equiv N_{bin}$ in Eqs. (S1.3) and (S1.5)) of non overlapping equal sized consecutive subintervals $A_k : [a, b] = \bigcup_{k=1}^{N_{bin}} A_k$ and $A_n \bigcap A_m = \emptyset \ \forall n \neq m$. Then, recourse to the usual histogram method, based on counting the relative frequencies of the time series' values within each subinterval, is made. Of course, in this approach the temporal order in which the time-series values emerge plays no role at all. The only pieces of information we have here are the x_t-values that allow one to assign inclusion within a given bin, ignoring just where they are located (this is, the subindex i). Note that the division procedure of the interval $[a, b]$ provides the natural order sequence for the evaluation of the PDF gradient involved in Fisher's information measure.

From now on, we assume that the all Information Theory quantifiers will be evaluated with BP-PDF's, by this reason usually they are called permutation quantifiers or time causal Information Theory quantifiers. In our current paper, we chose the lexicographic ordering given by the algorithm of Lehmer [23], among other possibilities, due to its better distinction of different dynamics in the Shannon–Fisher plane, $\mathcal{H} \times \mathcal{F}$ (see [21, 22]).

Causal information planes

In statistical mechanics one is often interested in isolated systems characterized by an initial, arbitrary, and discrete probability distribution. Evolution towards equilibrium is to be described, as the overriding goal. At equilibrium, we can suppose, without loss of generality, that this state is given by the equiprobable distribution $P_e = \{p_i = 1/N, \forall i = 1, \dots, N\}$. The temporal evolution of the above introduced Information Theory quantifiers, Shannon entropy $\mathcal{H}$, statistical complexity $\mathcal{C}$ and Fisher information measure $\mathcal{F}$, can be analyzed using a two-dimensional (2D) diagrams of the corresponding quantifiers versus time t. However, the second law of thermodynamics states that, for isolated systems, entropy grows monotonically with time ($d\mathcal{H}/dt \geq 0$) [44]. This implies that entropy $\mathcal{H}$ can be regarded as an arrow of time, so that an equivalent way to study the temporal evolution of these quantifiers is using the normalized entropy $\mathcal{H}$ as substitute for the time-axis.

Two causal information planes are defined (the term causality remembers the fact that temporal correlations between successive samples are taken into account through the Bandt and Pompe PDF recipe used to estimate both Information Theory quantifiers): *a)* The *causality entropy–complexity plane*, $\mathcal{H} \times \mathcal{C}$, is based only on global characteristics of the associated time series PDF (both quantities are defined in terms of Shannon entropies); while *b)* the *causality Shannon-Fisher plane*, $\mathcal{H} \times \mathcal{F}$, is based on global and local characteristics of the PDF. In the case of $\mathcal{H} \times \mathcal{C}$ the variation range is $[0, 1] \times [\mathcal{C}_{min}, \mathcal{C}_{max}]$ (with $\mathcal{C}_{min}$ and $\mathcal{C}_{max}$ the minimum and maximum statistical complexity values, respectively, for a given $\mathcal{H}$ value [20]), while in the causality plane $\mathcal{H} \times \mathcal{F}$ the range is $[0, 1] \times [0, 1]$.

These two diagnostic tools were shown to be particularly efficient to distinguish between the deterministic chaotic and stochastic nature of a time series since the permutation quantifiers have distinctive behaviors for different types of motion. According to the findings obtained by Rosso *et al.* [31, 22, 45], chaotic maps have intermediate entropy $\mathcal{H}$ and Fisher $\mathcal{F}$ values, while complexity $\mathcal{C}$ reaches larger values, very close to those of the limit. For regular processes, entropy and complexity have small values, close to zero, while the Fisher is close to one. Finally, totally uncorrelated stochastic processes are located in the planar location associated with $\mathcal{H}$ near one and, $\mathcal{C}$, $\mathcal{F}$ near to zero, respectively. It has also been found that $1/f^{\alpha}$ correlated stochastic processes with $1 \leq \alpha \leq 3$ are characterized by intermediate permutation entropy and intermediate statistical complexity values [31], as well as, intermediate low Fisher information [31, 22, 45]. Moreover, note that in both causal information planes the localization of these stochastic behavior look like a separation border with respect to chaotic, which are localized above of it. In addition, these two causal information planes have been profitably used to visualization and characterization of different dynamical regimes when the system parameters vary [21, 22, 46, 47, 48, 49, 50, 51, 52, 53, 54]; to study time dynamic evolution [39, 55, 56]; identifying periodicities in natural time series [57]; identification of deterministic dynamics contaminated with noise [32, 33]; estimating intrinsic time scales and delayed systems [40, 41, 42, 58]; characterization of pseudo-random number generators [59, 60] measure of complexity of two-dimensional patterns [61] among other biomedical and econophysics applications (see [43] and references therein).

References

[1] Gray, RM (1990) Entropy and Information Theory. Springer, Berlin-Heidelberg, Germany.

[2] Brissaud, JB (2005) The meaning of entropy. Entropy 7: 68–96.

[3] Shannon CE (1948) A mathematical theory of communication. Bell Syst. Technol. J. 27: 379–423, 623–56.

[4] Shannon, CE, Weaver, W (1949) The Mathematical Theory of Communication; University of Illinois Press: Champaign, IL, USA.

[5] Fisher, RA (1922) On the mathematical foundations of theoretical statistics. Philos. Trans. R. Soc. Lond. Ser. A 222: 309–368.

[6] Frieden RB (2004) Science from Fisher information: A Unification. Cambridge University Press, Cambridge, UK.

[7] Zografos K, Ferentinos K, Papaioannou T (1986) Discrete approximations to the Csiszár, Renyi, and Fisher measures of information. Canad. J. Stat. 14: 355–366 .

[8] Pardo L, Morales D, Ferentinos K, Zografos K (1994) Discretization problems on generalized entropies and R-divergences. Kybernetika 30: 445–460 .

[9] Sánchez-Moreno P, Dehesa JS, Yáñez RJ (2009) Discrete densities and Fisher Information. Proceedings of the 14th International Conference on Difference Equations and Applications. Uğur-Bahçeşehir University Publishing Company, Istanbul, Turkey Difference Equations and Applications: 291–298.

[10] Sánchez-Moreno P, Zarzo A, Dehesa JS (2012) Jensen divergence based on Fisher's information. Journal of Physics A: Mathematical and Theoretical. 45: 125305.

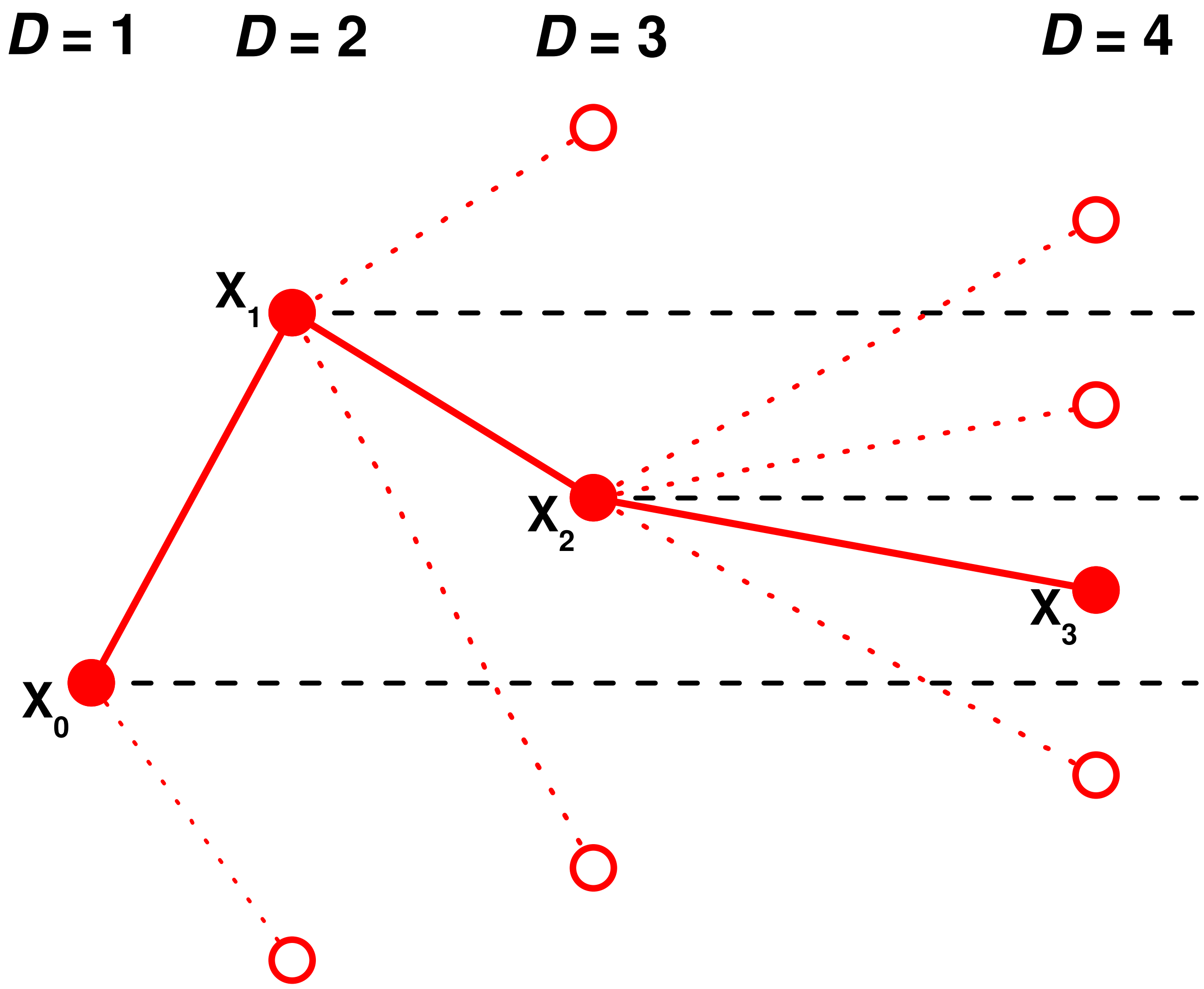

Figure A: Illustration of the construction principle for ordinal patterns of length D [38]. If $D = 4$ and $\tau = 1$, full circles and continuous lines represent the sequence of values $x_0 < x_1 > x_2 > x_3$ which lead to the pattern $\pi = [0321]$.

[11] Feldman DP, Crutchfield JP (1998) Measures of Statistical Complexity: Why? Phys. Lett. A 238: 244– 252.

[12] Feldman DP, McTague CS, Crutchfield JP (2008) The organization of intrinsic computation: Complexity-entropy diagrams and the diversity of natural information processing. Chaos 18: 043106.

[13] Kantz H, Kurths J, Meyer-Kress G (1998) Nonlinear Analysis of Physiological Data, Springer, Berlin.

[14] López-Ruiz R, Mancini HL, Calbet X (1995) A statistical measure of complexity. Phys. Lett. A 209: 321–326.

[15] Martín, MT, Plastino, A, Rosso, OA (2003) Statistical complexity and disequilibrium, Phys. Lett. A 311: 126–132.

[16] Lamberti PW, Martín MT, Plastino A, Rosso OA (2004) Intensive entropic non-triviality measure. Physica A 334: 119–131.

[17] Lin J (1991) Divergence measures based on the Shannon Entropy IEEE Transactions on Information Theory 37(1): 145–151.

[18] Grosse I, Bernaola-Galván P, Carpena P, Román-Roldán R, Oliver J, Stanley HE (2002) Analysis of symbolic sequences using the Jensen-Shannon divergence. Phys. Rev. E 65: 041905.

[19] Ré MA, Azad RK (2014) Generalization of entropy based divergence measures for symbolic sequence analysis. Plos ONE 9(4): e93532.

[20] Martín MT, Plastino A, Rosso OA (2006) Generalized statistical complexity measures: Geometrical and analytical properties. Physica A 369: 439–462.

[21] Olivares F, Plastino A, Rosso OA (2012) Ambiguities in the Bandt-Pompe's methodology for local entropic quantifiers. Physica A 391: 2518–2526.

[22] Olivares F, Plastino A, Rosso OA (2012) Contrasting chaos with noise via local versus global information quantifiers, Phys. Lett. A 376: 1577–1583.

[23] Schwarz, K (2011) The Archive of Interesting Code, `http://www.keithschwarz.com/interesting/code/?dir=factoradic-permutation`

[24] Vignat C, Bercher JF (2003) Analysis of signals in the Fisher–Shannon information plane. Phys. Lett. A 312: 27–33.

[25] Rosso OA, Craig H, Moscato P (2009) Shakespeare and other english renaissance authors as characterized by Information Theory complexity quantifiers. Physica A 388: 916–926.

[26] De Micco L, González CM, Larrondo HA, Martín MT, Plastino A, Rosso OA (2008) Randomizing nonlinear maps via symbolic dynamics. Physica A 387: 3373–3383.

[27] Mischaikow K, Mrozek M, Reiss J, Szymczak A (1999) Construction of symbolic dynamics from experimental time series. Phys. Rev. Lett. 82 1114–1147.

[28] Powell GE, Percival I (1979) A spectral entropy method for distinguishing regular and irregular motion of hamiltonian systems. J. Phys. A: Math. Gen. 12 2053–2071.

[29] Rosso OA, Blanco S, Jordanova J, Kolev V, Figliola A, Schürmann M, Başar E (2001) Wavelet entropy: a new tool for analysis of short duration brain electrical signals. J. Neurosc. Meth. 105 65–75.

[30] Bandt C, Pompe B (2002) Permutation Entropy: A Natural Complexity Measure for Time Series. Phys. Rev. Lett. 88: 174102.

[31] Rosso OA, Larrondo HA, Martín MT, Plastino A, Fuentes MA (2007) Distinguishing noise from chaos. Phys. Rev. Lett. 99: 154102.

[32] Rosso OA, Carpi LC, Saco PM, Gómez Ravetti M, Plastino A, Larrondo H (2012) Causality and the Entropy-Complexity Plane: Robustness and Missing Ordinal Patterns Physica A 391 42–55.

[33] Rosso OA, Carpi LC, Saco PM, Gómez Ravetti M, Larrondo H, Plastino A(2012) The Amigó paradigm of forbidden/missing patterns: a detailed analysis Eur. Phys. J. B 85: 419–430.

[34] Rosso OA, Olivares F, Zunino L, De Micco L, Aquino ALL, Plastino A, Larrondo HA (2013) Characterization of chaotic maps using the permutation Bandt-Pompe probability distribution. Eur. Phys. J. B 86: 116–129.

[35] Rosso OA, Masoller C (2009) Detecting and quantifying stochastic and coherence resonances via information-theory complexity measurements. Phys. Rev. E 79: 040106(R).

[36] Rosso OA, Masoller C (2009) Detecting and quantifying temporal correlations in stochastic resonance via information theory measures. Eur. Phys. J. B 69: 37–43.

[37] Saco PM, Carpi LC, Figliola A, Serrano E, Rosso OA (2010) Entropy analysis of the dynamics of El Niño/Southern Oscillation during the Holocene. Physica A 389: 5022–5027.

[38] Parlitz U, Berg S, Luther S, Schirdewan A, Kurths J, Wessel N (2012) Classifying cardiac biosignals using ordinal pattern statistics and symbolic dynamics. Comput. Biol. Med. 42: 319–327.

[39] Kowalski AM, Martín MT, Plastino A, Rosso OA (2007) Bandt-Pompe approach to the classical-quantum transition. Physica D 233: 21–31.

[40] Zunino L, Soriano MC, Fischer I, Rosso OA, Mirasso CR (2010) Permutation information-theory approach to unveil delay dynamics from time-series analysis. Phys. Rev. E 82: 046212.

[41] Soriano MC, Zunino L, Rosso OA, Fischer I, Mirasso CR (2011) Time scales of a chaotic semiconductor laser with optical feedback under the lens of a permutation information analysis. IEEE J. Quantum Electron. 47: 252–261.

[42] Zunino L, Soriano MC, Rosso OA (2012) Distinguishing chaotic and stochastic dynamics from time series by using a multiscale symbolic approach. Phys. Rev. E 86: 046210.

[43] Zanin M, Zunino L, Rosso OA, Papo D (2012) Permutation entropy and its main biomedical and econophysics applications: A review. Entropy 14: 1553–1577.

[44] Plastino AR, Plastino A (1996) Symmetries of the Fokker-Plank equation and Fisher-Frieden arrow of time. Phys. Rev. E 54: 4423 – 4326.

[45] Rosso OA, Olivares F, Plastino A (2015) Noise versus chaos in a causal Fisher-Shannon plane. Papers in Physics 7 070006.

[46] Rosso OA, De Micco L, Plastino A, Larrondo H (2010) Info-quantifiers' map-characterization revisited. Physica A 389: 249–262.

[47] Kowalski AM, Martín MT, Plastino A, Rosso OA (2011) Fisher-information description of the classical-quantal transition. Physica A 390:2435–2441.

[48] De Micco L, Fernández JG, Larrondo HA, Plastino A, Rosso OA (2012) Sampling period, statistical complexity, and chaotic attractors. Physica A 391: 2564–2575.

[49] Lange H, Rosso OA, Hauhs M (2013) Ordinal pattern and statistical complexity analysis of daily stream flow time series Eur. Phys. J. Special Topics 222: 535–552.

[50] Serinaldi F, Zunino L, Rosso OA (2014) Complexity-entropy analysis of daily stream flow time series in the continental United States Stochastic Environmental Research and Risk Assessment 28: 1685–1708.

[51] Montani F, Deleglise EB, Rosso OA (2014) Efficiency characterization of a large neuronal network: a causal information approach Physica A 401:58–70.

[52] Montani F, Rosso OA (2014) Entropy-Complexity Characterization of Brain Development in Chickens. Entropy 16: 4677–4692.

[53] Montani F, Rosso OA, Matias F, Bressler SL, Mirasso CR (2015) A symbolic information approach to determine anticipated and delayed synchronization in neuronal circuit models. Phil. Trans. R. Soc. A 373: 20150110.

[54] Montani F, Baravalle R, Montangie L, Rosso OA (2015) Causal information quantification of prominent dynamical features of biological neurons. Phil. Trans. R. Soc. A 373: 20150109.

[55] Bariviera A, Guercio MB, Martinez LB, Rosso OA (2015) The (in)visible hand in the Libor market: an Information Theory approach. Eur. Phys. J. B 88: 208.

[56] Bariviera A, Guercio MB, Martinez LB, Rosso OA (2015) A permutation Information Theory tour through different interest rate maturities: the LIBOR case. Phil. Trans. R. Soc. A 373: 20150119.

[57] Bandt C (2005) Ordinal time series analysis. Ecol. Modell. 182: 229–238.

[58] Aquino ALL, Cavalcante TSG, Almeida ES, Frery A, Rosso OA (2015) Characterization of vehicle behavior with information theory. Eur. Phys. J. B 85: 257.

[59] De Micco L, González CM, Larrondo HA, Martín MT, Plastino A, Rosso OA (2008) Randomizing nonlinear maps via symbolic dynamics. Physica A 387:3373–3383

[60] De Micco L, Larrondo HA, Plastino A, Rosso OA (2009) Quantifiers for randomness of chaotic pseudo-random number generators. Phil. Trans. R. Soc. A 367: 3281–3296.

[61] Ribeiro HV, Zunino L, Lenzi EK, Santoro PA, Mendes RS (2012) Complexity-Entropy Causality Plane as a Complexity Measure for Two-Dimensional Patterns. Plos One 7: e40689.
