# Supplementary material for: Classification and Verification of Handwritten Signatures with Time Causal Information Theory Quantifiers
Source: PLoS One. 2016 Dec 1;11(12):e0166868. doi: 10.1371/journal.pone.0166868 (PMC5131934; doi:10.1371/journal.pone.0166868)
Supplement: S2 File — (PDF) [file pone.0166868.s002.pdf]

Support Vector Machines and One-Class Support Vector Machines

Support Vector Machines (SVMs) were introduced by Vapnik and co-workers [1, 2, 3, 4], and extended by a number of other researchers. Their remarkably robust performance with respect to sparse and noisy data makes them the choice in several applications. A SVM is primarily a method that performs classification tasks by constructing hyperplanes in a multidimensional space that separates cases of different class labels. SVMs perform both regression and classification tasks and can handle multiple continuous and categorical variables. To construct an optimal hyperplane, a SVM employs an iterative training algorithm, which is used to minimize an error function.

One-Class Support Vector Machines (OC-SVMs) are a natural extension of SVMs [5, 6]. In order to identify suspicious observations, an OC-SVM estimates a distribution that encompasses most of the observations, and then labels as "suspicious" those that lie far from it with respect to a suitable metric. An OC-SVM solution is built estimating a probability distribution function which makes most of the observed data more likely than the rest, and a decision rule that separates these observation by the largest possible margin. The computational complexity of the learning phase is intensive because the training of an OC-SVM involves a quadratic programming problem [2], but once the decision function is determined, it can be used to predict the class label of new test data effortlessly.

In our case, the observations are six-dimensional vectors: Entropy, Complexity and Fisher Information in each of the two directions, horizontal and vertical, and we train the OC-SVM with genuine signatures. Let $\mathcal{Z} = \{z_1, z_2, \ldots, z_N\}$ be the six-dimensional training examples of genuine signatures. Let $\Phi\colon \mathcal{Z} \to \mathcal{G}$ be a kernel map which transforms the training examples to another space. Then, to separate the data set from the origin, one needs to solve the following quadratic programming problem:

$$\min_{\mathbf{w}\in\mathcal{G},\xi_i,b\in\mathbb{R}} \quad \left\{ \frac{1}{2}\|\mathbf{w}\|^2 + \frac{1}{\nu N}\sum_{i=1}^{N} \xi_i - b \right\} \tag{S2.1}$$

subject to

$$\nu \in (0,1],\ \xi_i \geq 0,\ \forall i = 1, \ldots, N, \text{ and} \tag{S2.2}$$

$$(\mathbf{w}\cdot\Phi(z_i)) \geq b - \xi_i,\ \forall i = 1, \ldots, N, \tag{S2.3}$$

where ξ_i are nonzero slack variables which allow the procedure to incur in errors. The parameter ν characterizes the solution as *a)* it sets an upper bound on the fraction of outliers (training examples regarded out-of-class) and, *b)* it is a lower bound on the number of training examples used as Support Vectors. We used $\nu = 0.1$ in our proposal.

Using Lagrange techniques and a kernel function $K(z, z_i) = \Phi(z)^T\Phi(z_i)$, for the dot-product calculations, the decision function $f(z)$ becomes:

$$f(z) = \operatorname{sign}\left\{(\mathbf{w}\cdot\Phi(z)) - b\right\} = \operatorname{sign}\left\{\sum_{i=1}^{N} \alpha_i\ K(z, z_i) - b\right\}. \tag{S2.4}$$

This method thus creates a hyperplane characterized by $\mathbf{w}$ and b which has maximal distance from the origin in the feature space $\mathcal{G}$ and separates all the data points from the origin. Here α_i are the Lagrange multipliers; every $\alpha_i > 0$ is weighted in the decision function and thus "supports" the machine; hence the name Support Vector Machine. Since SVMs are considered to be sparse, there will be relatively few Lagrange multipliers with a nonzero value.

Our choice for the kernel is the Gaussian Radial Base function:

$$K(z_i, z_j) = \exp\Big(-\frac{1}{2\sigma^2}\|z_i - z_j\|^2\Big), \tag{S2.5}$$

where $\sigma \in \mathbb{R}$ is a kernel parameter and $\|z_i - z_j\|^2$ is the dissimilarity measure; we used Euclidean distance.

The parameter $\sigma^2 = 10$ was selected by 5-fold-cross validation, that its, the dataset is divided into five disjoint subsets, and the method is repeated five times. Each time, one of the subsets is used as the test set and the other four subsets are put together to form the training set. Then the average error across all trials is computed. Every observation belongs to a test set exactly once, and belongs to a training set four times. Accuracy (ACC), Area Under the ROC Curve (AUC) and Equal Error Rate (EER) are used as performance measures [7].

In the context of signature verification one-class classification problems, a false positive occurs when a genuine signature is erroneously classified as being atypical. The probability of false positive misclassification is the false positive rate, which is controlled by the parameters ν in the aforementioned OC-SVM formulation. The parameter ν can be fixed a *priori* and it corresponds to the percentage of observations of the typical data which will be assigned as the Type I Error.

The `R` interface to `libsvm` in package `e1071` is designed to be as intuitive as possible. In the following we generate a toy dataset in $\mathbb{R}^2$, and show how to train and test an SVM classifier.

```
n <- 150 # number of data points
p <- 2 # dimension
sigma <- 1 # variance of the distribution
meanpos <- 0 # centre of the distribution of true signatures
meanneg <- 3 # centre of the distribution of false signatures
npos <- round(n/2) # number of true signatures
nneg <- n-npos # number of false signatures
# Generate the true and false signatures
xpos <- matrix(rnorm(npos*p, mean=meanpos, sd=sigma),npos,p)
xneg <- matrix(rnorm(nneg*p, mean=meanneg, sd=sigma),npos,p)
x <- rbind(xpos, xneg)
# Generate the labels of signatures
y <- matrix(c(rep(1, npos),rep(-1, nneg)))
```

Now we split the data into a training set (80%) and a test set (20%)

```
## Prepare a training and a test set ##
ntrain <- round(n*0.8) # number of training examples
tindex <- sample(n,ntrain) # indices of training samples
xtrain <- x[tindex,]; xtest <- x[-tindex,]
ytrain <- y[tindex];  ytest <- y[-tindex]
istrain=rep(0,n); istrain[tindex]=1
```

Training a SVM (one-class) using the radial basis function kernel with fixed hyper-parameters $\nu = 0.1$ and $\sigma = 0.05$:

```
library(e1071)# Functions for support vector machines
library(rpart) # Recursive Partitioning and Regression Trees
```

```
svm.model <- svm(Type ~ ., data = trainset, type='one-classification',
nu=0.10, scale=TRUE, kernel="radial", gamma=0.05)
```

Prediction of class signatures

```
svm.pred <- predict(svm.model, testset[,-10])
```

For more details, see the Reproducible research material available at `http://www.de.ufpe.br/~raydonal/ReproducibleResearch/Signatures/Signatures.html`

References

[1] Campbell C, Ying Y. Learning with Support Vector Machines. In: Brachman RJ, Dietterich T, editors. Synthesis Lectures on Artificial Intelligence and Machine Learning. No. 5 in Synthesis Lectures on Artificial Intelligence and Machine Learning. Santa Fe, CA: Morgan and Claypool; 2011. p. 1–95.

[2] Boser BE, Guyon IM, Vapnik VN. A training algorithm for optimal margin classifiers. In: Proceedings of the Fifth Annual Workshop on Computational Learning Theory. Pittsburgh: ACM Press; 1992. p. 144–152.

[3] Vapnik VN. The Nature of Statistical Learning Theory. Springer; 1995.

[4] Vapnik VN. Statistical Learning Theory. Willey; 1998.

[5] Schölkopf B, Platt JC, Shawe-Taylor JC, Smola AJ, Williamson RC. Estimating the Support of a High-Dimensional Distribution. Neural Computation. 2001; 13(7): 1443–1471.

[6] Schölkopf B, Smola AJ. Learning with Kernels. Cambridge: MIT Press; 2002.

[7] Provost F, Kohavi R. On Applied Research in Machine Learning. Machine Learning. 1998; 30(2/3): 127–132.
