# Supplementary material for: Classification and Verification of Handwritten Signatures with Time Causal Information Theory Quantifiers
Source: PLoS One. 2016 Dec 1;11(12):e0166868. doi: 10.1371/journal.pone.0166868 (PMC5131934; doi:10.1371/journal.pone.0166868)
Supplement: S3 File — (PDF) [file pone.0166868.s003.pdf]

# Exploratory data analysis

Figs A, B, C, and D present the supplementary exploratory data analysis of those features that were not presented in the body of the article.

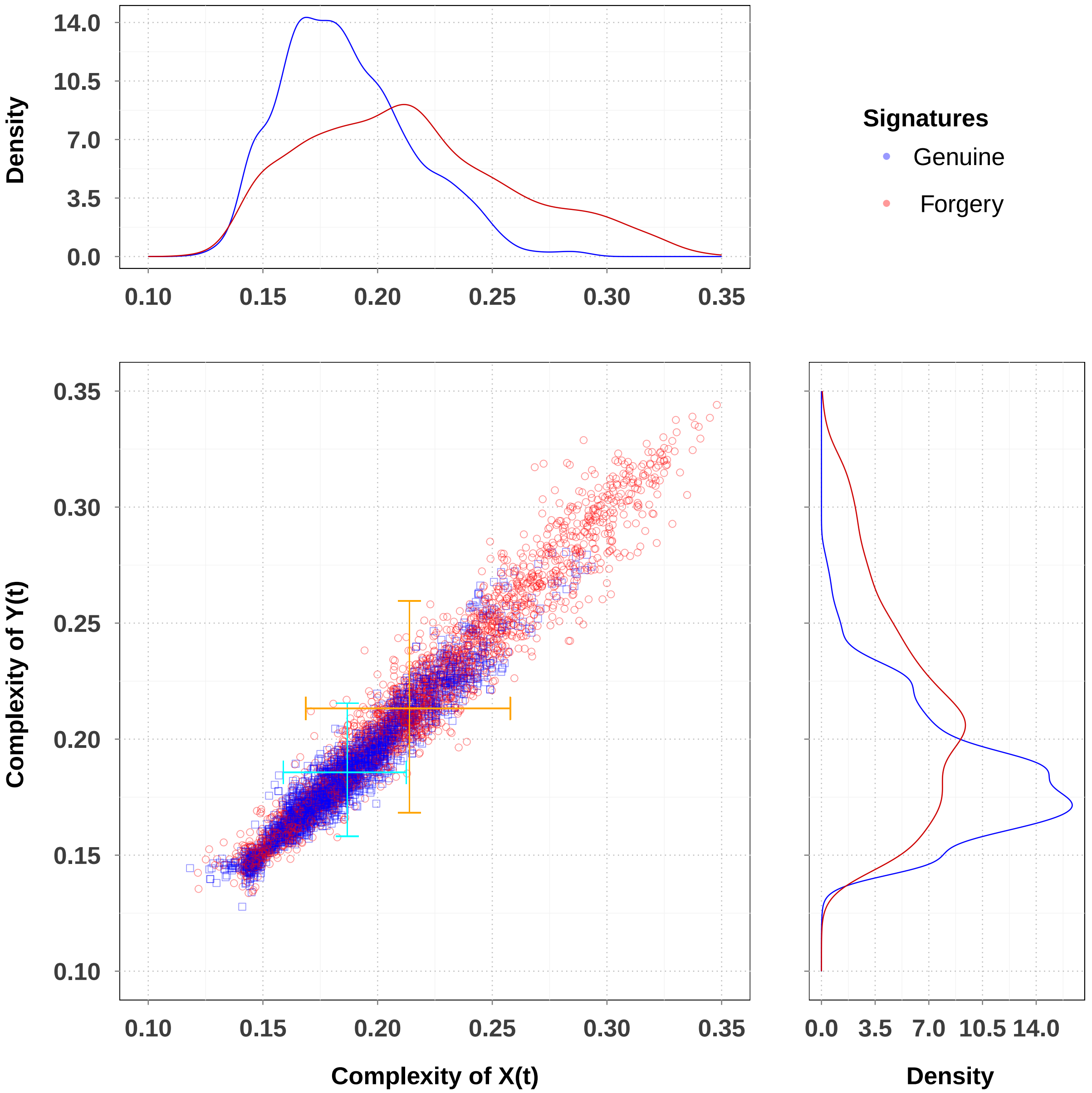


Figure A: Scatter plot with marginal kernel density estimates of complexity quantifiers in both trajectory coordinates time series **X** and **Y**. Genuine (blue) and skilled forgery signatures (red points), 100 subjects. Marginal kernel densities depict the distribution of statistical complexity along both axes.

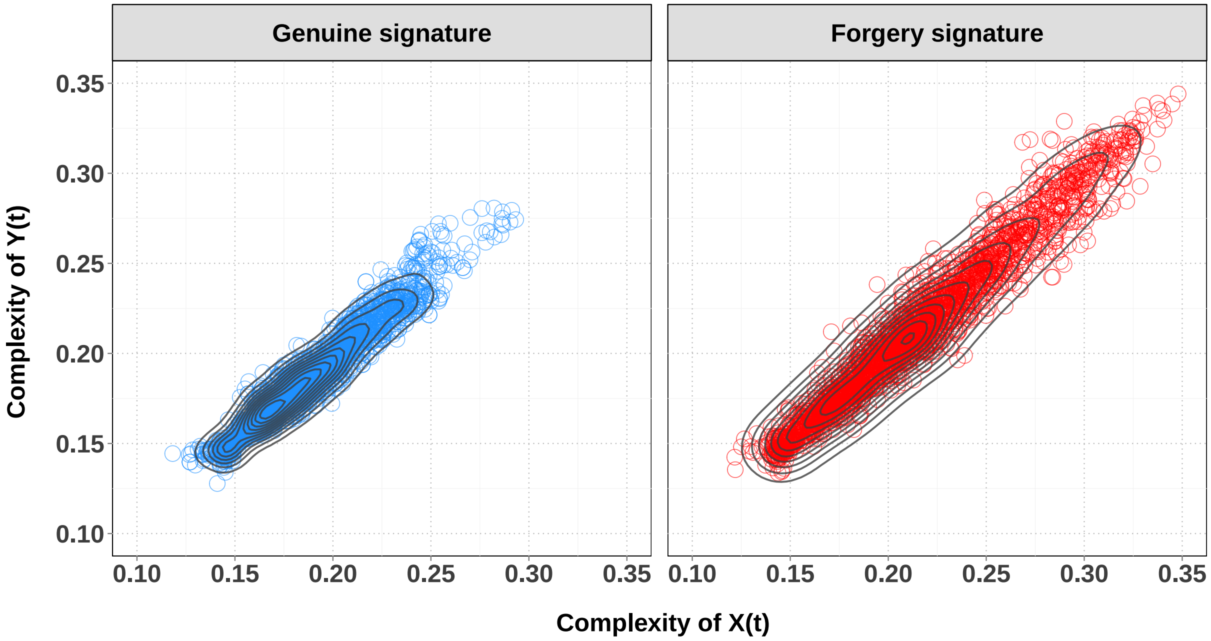


Figure B: Contour plot superimposed on the scatterplot of statistical complexity for genuine (right panel) and skilled forgery signatures (left panel).

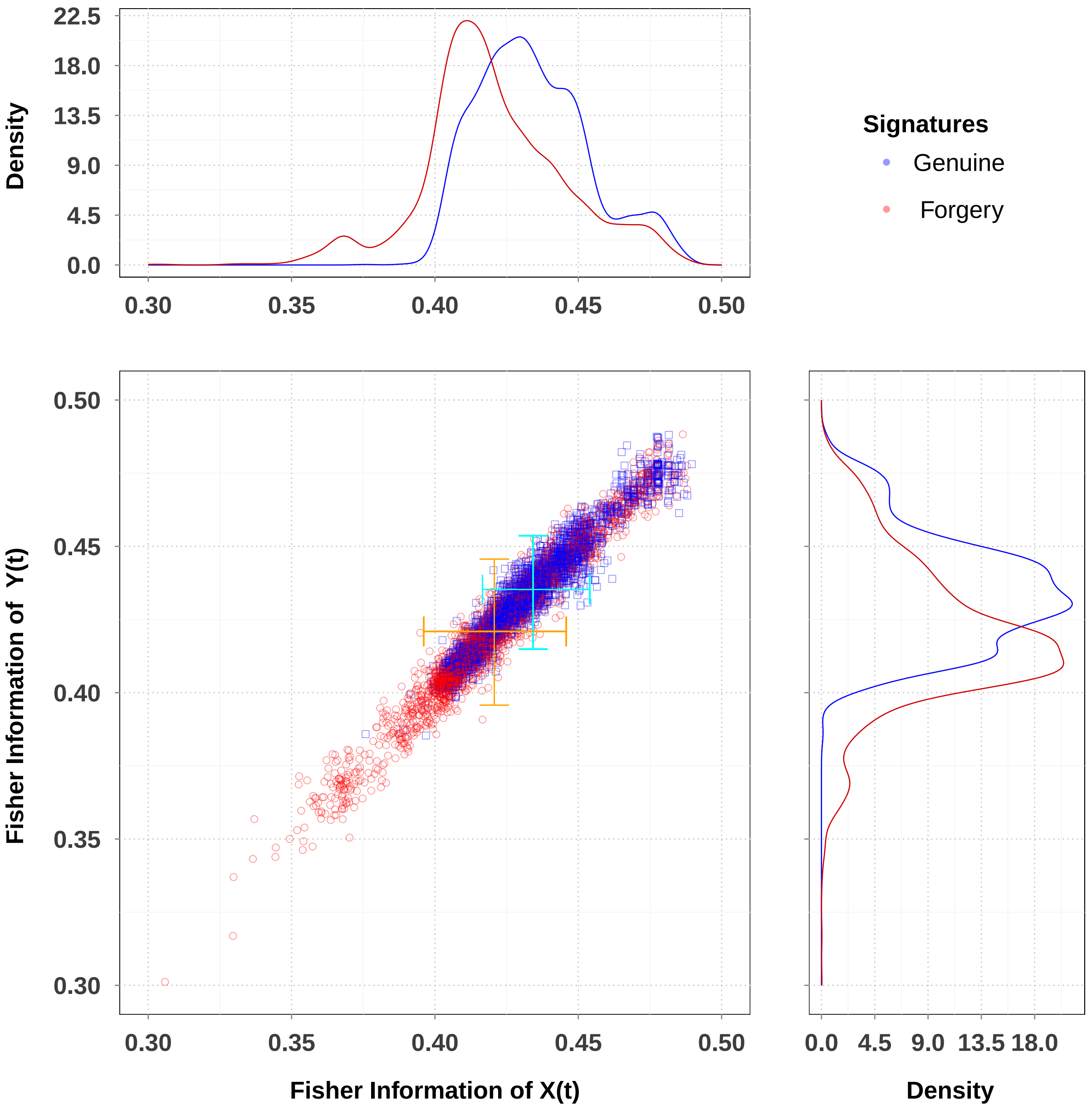


Figure C: Scatter plot with marginal kernel density estimates of Fisher information quantifiers in both trajectory coordinates time series **X** and **Y**. Genuine (blue) and skilled forgery signatures (red points), 100 subjects. Marginal kernel densities depict the distribution of Fisher information along both axes.

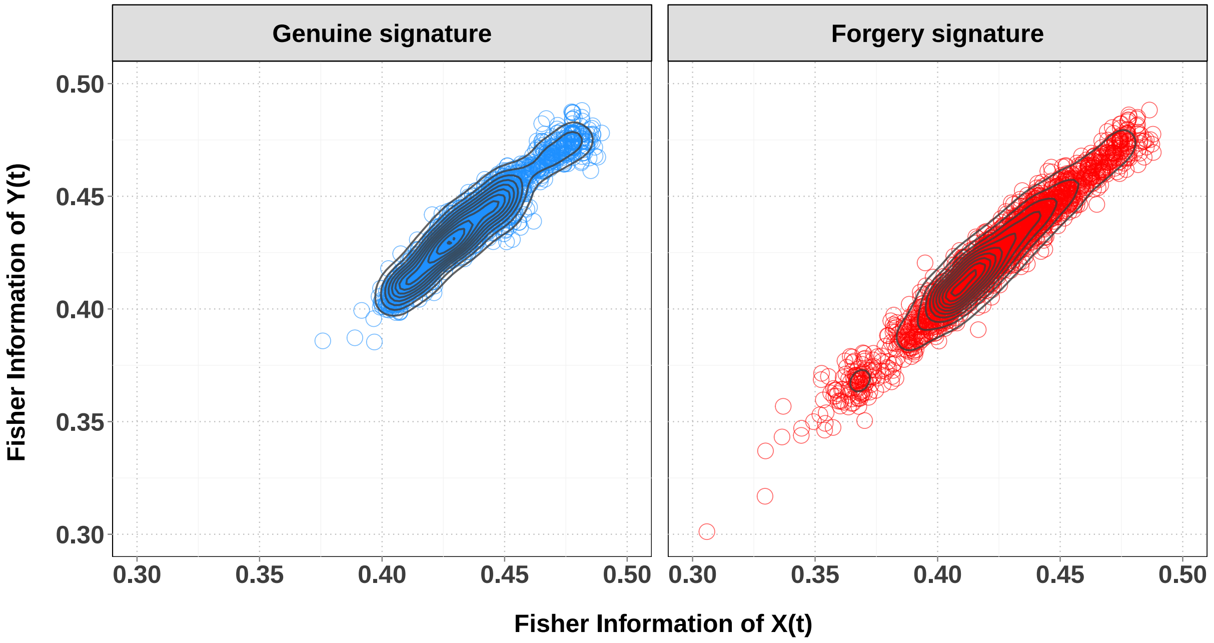


Figure D: Contour plot superimposed on the scatterplot of Fisher information quantifiers for genuine (right panel) and skilled forgery signatures (left panel).
