# Supplementary material for: Classification and Verification of Handwritten Signatures with Time Causal Information Theory Quantifiers
Source: PLoS One. 2016 Dec 1;11(12):e0166868. doi: 10.1371/journal.pone.0166868 (PMC5131934; doi:10.1371/journal.pone.0166868)
Supplement: S4 File — (PDF) [file pone.0166868.s004.pdf]

Signature classification

Figs A and B present the circular dendrograms of the two additional features: statistical complexity and Fisher information.

Figs C and D show the classification by the rule of the parallelepiped of genuine signatures using statistical complexity and Fisher information obtained as result of the application of entropy classification.

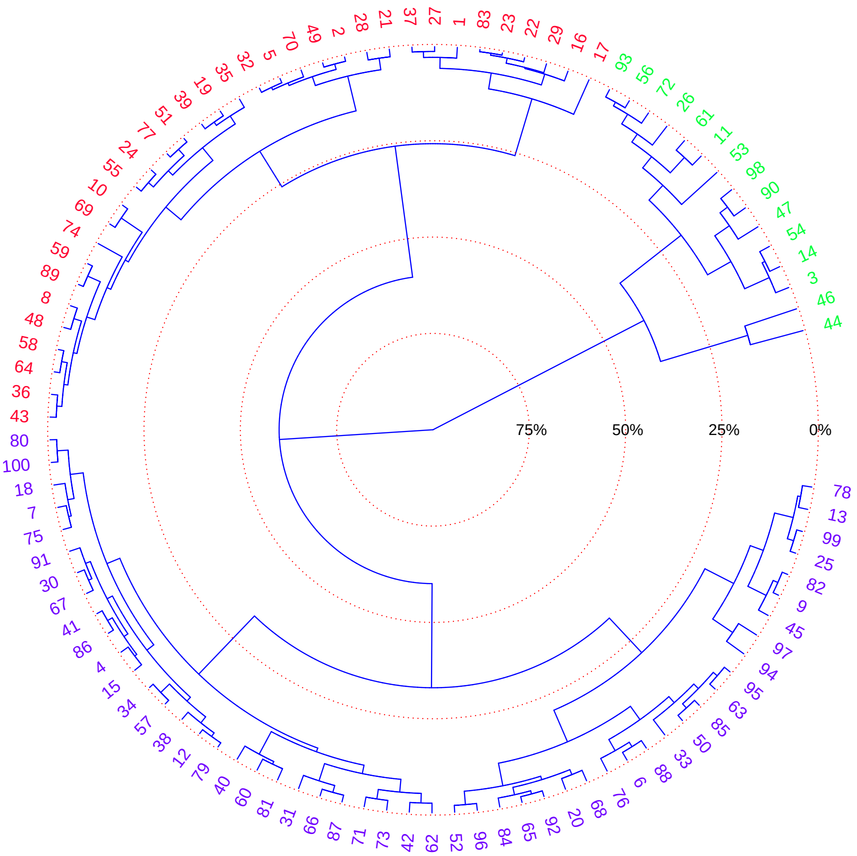

Figure A: Neighbor-joining, rooted, circular dendrogram clustering of genuine signatures by statistical complexity: H1, H2, and H3, in red, blue, and green, respectively.

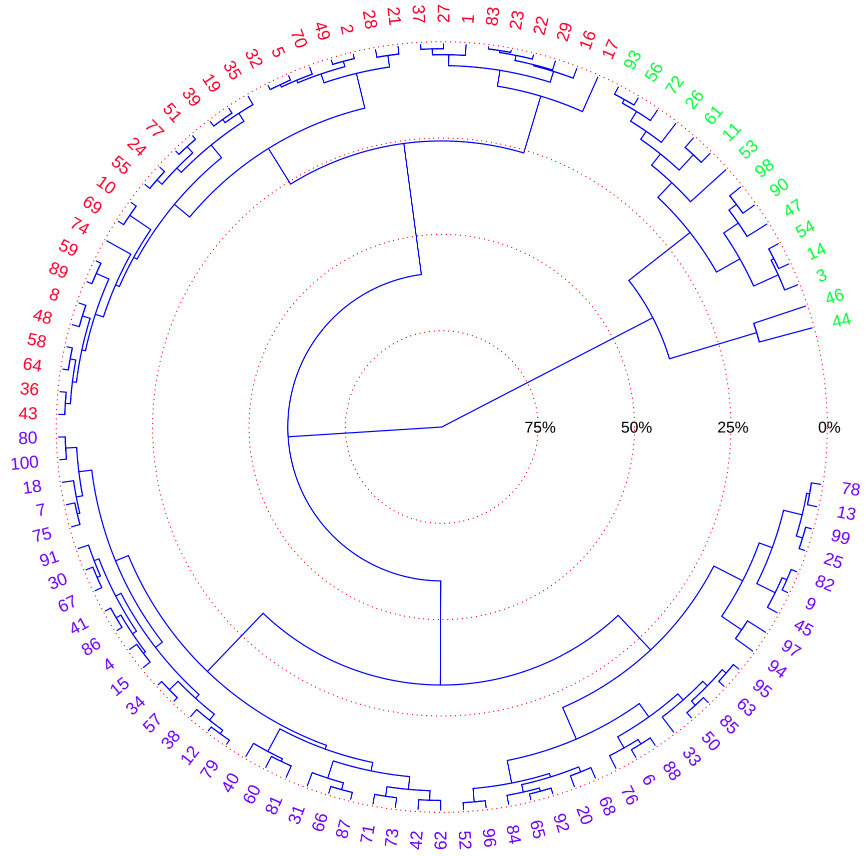

Figure B: Neighbor-joining, rooted, circular dendrogram clustering of genuine signatures by Fisher information: H1, H2, and H3, in red, blue, and green, respectively.

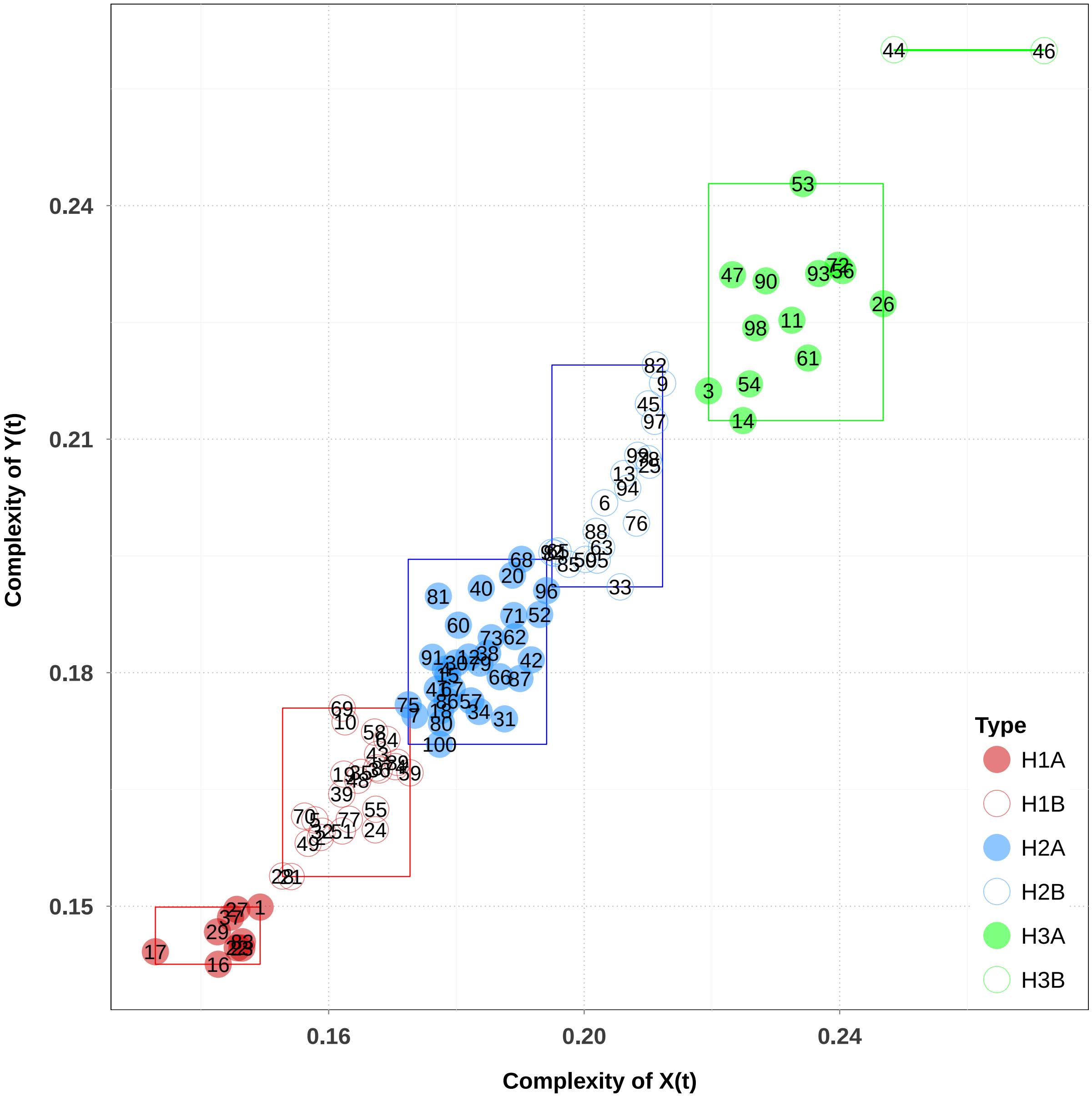

Figure C: Classification by the rule of the parallelepiped of genuine signatures using statistical complexity obtained as result of the application of entropy classification. Each subject is identified by its ID.

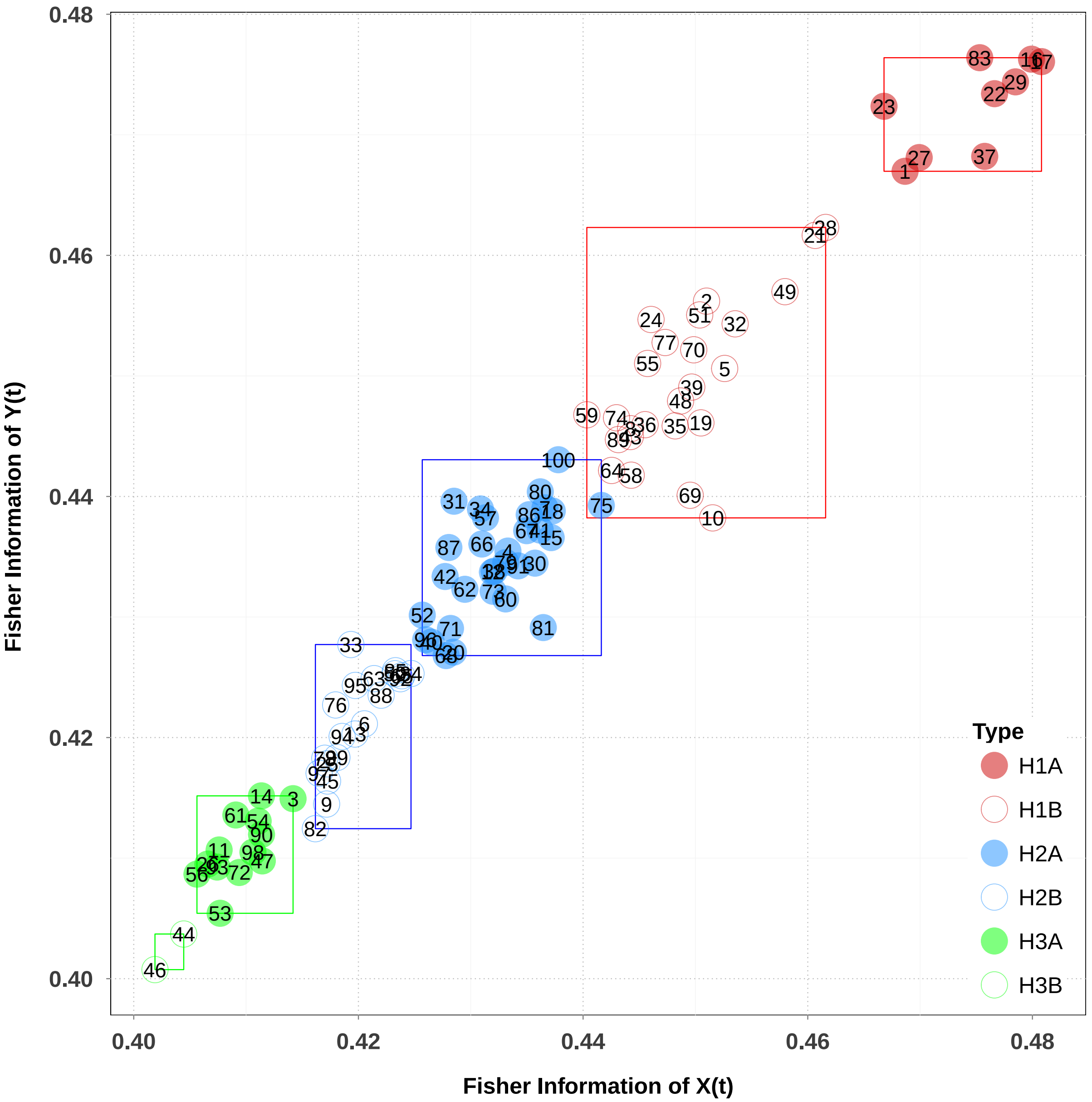

Figure D: Classification by the rule of the parallelepiped of genuine signatures using Fisher information obtained as result of the application of entropy classification. Each subject is identified by its ID.
